# Remote ischemic preconditioning protects against spinal cord ischemia–reperfusion injury in mice by activating NMDAR/AMPK/PGC-1α/SIRT3 signaling

**DOI:** 10.1186/s13578-023-00999-4

**Published:** 2023-03-16

**Authors:** Changjiang Gu, Fanqi Kong, Junkai Zeng, Xiangwu Geng, Yanqing Sun, Xiongsheng Chen

**Affiliations:** 1 Spine Center, Department of Orthopaedics, Changzheng Hospital, Naval Medical University (Second Military Medical University), Shanghai, 200003 People’s Republic of China; 2 Department of Orthopaedics, Shanghai General Hospital, Shanghai Jiao Tong University School of Medicine, 85 Wujin Road, 200080 Shanghai, PR China

**Keywords:** Oxidative stress, RIPC, SIRT3, Ischemia reperfusion injury

## Abstract

**Background:**

To study the protective effects of delayed remote ischemic preconditioning (RIPC) against spinal cord ischemia–reperfusion injury (SCIRI) in mice and determine whether SIRT3 is involved in this protection and portrayed its upstream regulatory mechanisms.

**Methods:**

In *vivo*, WT or SIRT3 global knockout (KO) mice were exposed to right upper and lower limbs RIPC or sham ischemia. After 24 h, the abdominal aorta was clamped for 20 min, then re-perfused for 3 days. The motor function of mice, number of Nissl bodies, apoptotic rate of neurons, and related indexes of oxidative stress in the spinal cord were measured to evaluate for neuroprotective effects. The expression and correlation of SIRT3 and NMDAR were detected by WB and immunofluorescence. In vitro*,* primary neurons were exacted and OGD/R was performed to simulate SCIRI in vivo. Neuronal damage was assessed by observing neuron morphology, detecting LDH release ratio, and flow cytometry to analyze the apoptosis. MnSOD and CAT enzyme activities, GSH and ROS level were also measured to assess neuronal antioxidant capacity. NMDAR-AMPK-PGC-1α signaling was detected by WB to portray upstream regulatory mechanisms of RIPC regulating SIRT3.

**Results:**

Compared to the SCIRI mice without RIPC, mice with RIPC displayed improved motor function recovery, a reduced neuronal loss, and enhanced antioxidant capacity. To the contrary, the KO mice did not exhibit any effect of RIPC-induced neuroprotection. Similar results were observed in vitro. Further analyses with spinal cord tissues or primary neurons detected enhanced MnSOD and CAT activities, as well as increased GSH level but decreased MDA or ROS production in the RIPC + I/R mice or NMDA + OGD/R neurons. However, these changes were completely inhibited by the absence of SIRT3. Additionally, NMDAR-AMPK-PGC-1α signaling was activated to upregulate SIRT3 levels, which is essential for RIPC-mediated neuroprotection.

**Conclusions:**

RIPC enhances spinal cord ischemia tolerance in a SIRT3-dependent manner, and its induced elevated SIRT3 levels are mediated by the NMDAR-AMPK-PGC-1α signaling pathway. Combined therapy targeting SIRT3 is a promising direction for treating SCIRI.

**Supplementary Information:**

The online version contains supplementary material available at 10.1186/s13578-023-00999-4.

## Introduction

Spinal cord ischemia–reperfusion injury (SCIRI) is a major cause of paraplegia following surgery on the spine and thoracic aorta, resulting in high morbidity and reduced quality of life [1–4]. Although surgical advances have reduced the risk of postoperative paraplegia, the prevention of SCIRI remains a major problem in thoracic aorta surgery and spine surgery.

Remote ischemic preconditioning (RIPC), which is characterized by short episodes of sub-lethal ischemia in distant-tissue, protects against prolonged ischemia in target organs, has potential for clinical application [5, 6]. The protective effect of RIPC against ischemia reperfusion (I/R) injury was first identified in the heart [7] and then progressively reported in other organs [8–11], including the spinal cord [5, 12–14]. There are two therapeutic time windows for RIPC-mediated protection against ischemic: the first occurs immediately after preconditioning and is called rapid ischemic tolerance, while the second, occurs 24–72 h after pretreatment, is called delayed ischemic tolerance [15, 16]. The protective mechanisms of delayed ischemic tolerance involved the activation of various receptors and transcription factors, as well as the upregulation of various cytoprotective genes and proteins [17]. However, the mechanisms, especially the key proteins, that mediate the protective effect of delayed ischemic tolerance remain unclear.

SIRT3 is a classical deacetylase mainly localized in mitochondria [18, 19], which maintains mitochondrial homeostasis by regulating oxidative stress of a variety of important antioxidant enzymes, such as manganese-dependent superoxide dismutase (MnSOD) and catalase (CAT) [20, 21]. It also controls reduced glutathione (GSH) levels through deacetylation [22]. The expression of SIRT3 has been reported to be down-regulated in a variety of organs during ischemia–reperfusion injury, and increased SIRT3 expression contribute to cell survival [18, 19, 23]. Emerging evidence indicates that RIPC improves ischemic tolerance by enhancing the antioxidant capacity of the spinal cord [24–27]. However, whether SIRT3 is involved in RIPC-mediated enhancement of spinal cord ischemia tolerance has not been reported. Here, we hypothesized that RIPC enhances neuronal antioxidant signaling and improves spinal cord ischemia tolerance by increasing SIRT3 expression.

Current research identifies moderate ischemia-induced hypoxic stress, acid stress and excitatory amino acid stress as common humoral factors in RIPC-induced neuroprotective effects, which result in the activation of transcription factors and the synthesis of protective genes and proteins for neurons [28]. Glutamate stress is unique in nervous system ischemia. Ischemia in the spinal cord induces membrane depolarization, which results in a massive glutamate release into the extracellular space. Overactivation of glutamate receptors, particularly NMDA receptors (NMDAR), result in a massive calcium influx, which in turn activates several cell injury processes [28]. However, NMDAR was also thought to be involved in ischemic tolerance, as NMDA receptor antagonists interfere with the protective effect of sub-lethal NMDA when administered before preconditioning [29, 30]. In a recent study, Mukai et al. discovered that glutamate concentrations in the ventral horn of the spinal cord were mildly elevated after RIPC and activated the neuronal surface NMDAR, and inhibition of NMDAR reduced the neuroprotective effects of RIPC [12]. NMDA receptors have also been shown to play a role in the mechanism of brain protection by ischemic preconditioning (IPC) [31–33]. However, the downstream mechanism by which NMDAR exerts its protective effect on neurons has not been elucidated.

In the present study, we investigated the role of SIRT3 in RIPC-induced spinal cord ischemic tolerance and examined the relationship between NMDAR and SIRT3 after RIPC. Through a series of in vivo and in vitro experiments, SIRT3 was identified as the key protein mediating the neuroprotective effect of RIPC. The sublethal activation of NMDAR may be involved in the elevation of SIRT3 level after RIPC through AMPK-PGC-1α signaling. We also explored a RIPC-based combined treatment therapy for SCIRI and exhibit promising efficacy and safety.

## Materials and methods

### Spinal cord ischemia–reperfusion injury in mice

All experiments were performed on adult male C57BL/6 J mice (20–25 g). Second Military Medical University Animal Care Committee gave the permission to all experiments. All the procedures were in accordance with the guidelines of the National Institute of Health Guide for the Care and Use of Laboratory Animals. The mouse SCIRI model was established as previously described [24, 25]. Briefly, the abdominal aorta was exposed at the level of the left renal artery through a 2–3 cm medial incision, a bull-dog clamp is used to clamp the aorta just below the renal artery to induce Spinal cord ischemia. After obstruction of blood flow lasted 20 min, the bulldog clamp was removed and the antibiotic (Gentamicin) was administered intramuscularly immediately. During spinal cord ischemia or reperfusion, mice body temperature or distal arterial blood pressure and heart rate were monitored and kept stable. Heparin (10 units) was administered 5 min before aortic occlusion and 0.25% (w/v) bupivacaine was applied around the wound for postoperative analgesia. Animals were housed separately after surgery and survived for 3 days. Bladder content was compressed manually as required.

### Remote ischemic preconditioning

RIPC was performed as previously reported [34]. In our model, RIPC was performed 1 day before SCIRI. It consisted of 4 cycles of ischemia in the right upper and lower extremities for 5 min followed by 5 min of reperfusion using tourniquets to interrupt blood flow. The skin changing color to cyanosis indicated the completion of limb ischemia.

### Neurologic and histopathologic evaluations

On the third day after reperfusion, the animals were assessed neurologically by two observers who were unaware of the grouping.

*The Basso mouse scale (BMS)* was conducted at 6 h, 12 h, 24 h, 48 h and 72 h after reperfusion to evaluate neurological function. Scores ranged from 0 (complete paraplegia) to 9 (normal function), 1–8 points represent varying degrees of neurological deficits [35].

*The footprint analysis* was performed on post-operative day 3 to assess the recovery of hindlimb coordination ability. As previously reported [36], blue and red dyes were applied to the front and hind paws of the mice. Then, the mice were encouraged to walk in a straight line on white paper. The pictures obtained were photographed and analyzed.

*Motor evoked potential (MEP)* amplitude was measured to assess motor functions of mice on the 3th day after injury. According to previous studies [37], Stimulation electrodes were applied to the surgically exposed anastomotic end of the spinal cord, recording electrodes were placed at the biceps femoris flexor, reference electrodes were placed at the distal tendons of the hind limb muscles and ground electrodes were placed subcutaneously. Peak-to-peak amplitude was used to indicate nerve conduction function in the mouse hind limb.

### Nissl staining

A histopathologic evaluation was performed in the spinal cord at 3 days after reperfusion. After trans-cardiac perfusion and fixation with 50 mL of 0.9% saline solution and 4% paraformaldehyde respectively, the spinal cord of L5 to L7 segments was removed and postfixed in the same fixative overnight. The spinal cord tissues were embedded in optimum cutting temperature compound (Sakura, CA, USA) and cut into serial transverse sections to a thickness of 5 μm after sucrose gradient dehydration. Three representative sections of each group were subjected to Nissl staining with 0.5% toluidine blue for histopathologic evaluation. The remaining intact motor neurons in the ischemic ventral anterior horn of the spinal cord in each animal were counted and averaged. Cells that contained Nissl substance in the cytoplasm, loose chromatin, and prominent nucleoli were considered normal motor neurons. To reduce counting bias, cell counting was performed by two independent investigators blinded to treatment history.

### TUNEL assay

The TUNEL assay was conducted using the TUNEL detection kit (Beyotime, shanghai, China) according to the instructions. Briefly, after fixation, permeabilization and blocking, spinal cord slices were stained with TUNEL reaction solution containing terminal deoxynucleotidyl transferase at 37 °C for 1 h. After washing with PBS for three times, the slices were co-stained with DAPI and visualized under a fluorescence microscope. The percentage of TUNEL positive cells in all cells was analyzed to assess apoptosis.

### Measurement of SOD, CAT activities, and GSH level

The enzyme activities of superoxide dismutase (SOD), catalase (CAT), and reduced-glutathione (GSH) level in tissues or cells were determined by using Cu/Zn-SOD and Mn-SOD Assay Kit with WST-8, Catalase Assay Kit, and GSH and GSSG Assay Kit (Beyotime, Shanghai, China) according to the manufacturer's instructions.

### Malondialdehyde (MDA) detection

MDA quantification was performed through thiobarbituric acid reactive substances (TBARS) assay using a lipid peroxidation MDA assay kit (Beyotime, Shanghai, China). In brief, after centrifugation of the tissue homogenate, the supernatant was added to the MDA assay working solution prepared according to the kit instructions and heated in a boiling water bath for 15 min. It was subsequently cooled on ice and centrifuged. 200 µl of the supernatant was added to a 96-well plate and the absorbance was measured at 532 nm using an enzyme marker.

### Primary neuron culture

Primary neurons were cultured as previously described [36]. Briefly, the brains from newborn mice (1–3 days old) were cut into 0.5–1 mm^3^ pieces and digested with 0.25% trypsin–EDTA solution (Thermo Fisher Scientific, MA, USA) for 15 min under gentle shaking. After termination of the reaction, the digested brain pieces were centrifuged at 1200r.p.m for 5 min and resuspended in DMEM/F12 (Gibco Laboratory) to acquire the cell suspensions. After passed through a nylon mesh (70-mm pore diameter), dissociated neurons were seeded on poly-D-lysine-coated plates. The medium was replaced with Neurobasal medium (Thermo Fisher Scientific) supplemented with 2% B27 (Gibco Laboratory, Grand Island, NY), 2 mM glutamine (Gibco) and 1% penicillin–streptomycin 4 h later. Half the culture medium was exchanged every 2 days.

### Oxygen glucose deprivation and reperfusion model

The OGD/R model was established to simulate SCIRI in vitro as previously described [38]. The primary neurons were cultured in sugar-free medium and placed in a modular incubator chamber that was flushed with 2L/min of a 95% N2/5% CO_2_ gas mixture for 15 min to remove oxygen. The chamber was then sealed and placed in a 37 °C incubator for 60 min. Then the medium was replaced with normal medium and cells were placed in a normal incubator for 12 h. Neuronal Pretreatment was induced by incubation for 4 h in growth medium containing 100 μM NMDA or 10 μM Glutamate as previously reported [30, 39].

### Cell viability assay

Cell viability was assessed using the Cell Counting Kit-8 (CCK-8) assay (Dojindo, Kumamoto, Japan). Primary neurons were plated in 96-well plate and pretreated with or without OGD treatment, and different concentrations of HKL were added. 10μL of CCK-8 solution was added to each well 12 h later and incubated at 37 °C with 5% CO_2_ for 4 h. The absorbance was measured at 450 nm using an absorbance microplate reader (ELx800; Bio-Tek, USA) to assess cell viability. Each group was repeated at least three times for analysis.

### LDH detection

Assessment of cell injury was done by determination of LDH activity released into the culture media with the reagent kit from Beyotime (Shanghai, China). Briefly, the culture media of neurons were collected after exposure to OGD/R and then the supernatant was obtained by centrifugation. In addition, the cells were lysed with 2% Triton for 1 h and centrifuged to remove cellular debris. After that, the culture supernatant and cell lysates were respectively incubated with LDH reaction mixture at 37 °C for 15 min. The absorbance was measured at 490 nm when the reaction was stopped, and LDH release was expressed as a percentage of total LDH.

### Neuron apoptosis assay

An annexin V-fluorescein isothiocyanate (FITC)/propidium iodide (PI) cell apoptosis kit (Thermo Fisher Scientific, MA, USA) was used to detect neuron apoptosis. After exposure to OGD/R, the primary neurons were collected and washed twice with cold phosphate buffered saline (PBS). Then the cells were resuspended in binding buffer and then incubated with FITC-labeled Annexin V and PI in the dark for 15 min after centrifugation at 1500 rpm for 5 min. FACScan flow cytometry (BD Biosciences) was used to analyze the apoptotic neurons.

### Intracellular ROS evaluation

The oxidation-sensitive fluorescent probe DCFH-DA (Beyotime, Shanghai, China) was used to measure the level of ROS in cells. Briefly, after specific treatment, cells were collected and incubated with DCFH-DA for 30 min and then washed three times with serum-free medium. The fluorescent signal intensity of DCF was analyzed by flow cytometry at 488 nm and 525 nm for excitation and emission, respectively.

### Intracellular Ca^2+^ concentration assessment

The intracellular Ca^2+^ level was measured according to the manufacturer’s instruction. Briefly, after glutamate exposure, neurons were collected and incubated in 5 μM Fluo-3 AM (Beyotime, Shanghai, China) for 40 min at 37 °C without light. Then cells were washed with Hank's solution and incubated for additional 20 min at 37 °C to ensure that Fluo-3 AM was completely transformed into cells. Finally, the fluorescence intensity of intracellular Fluo-3 was assessed by flow cytometry.

### Neuronal lentiviral particle transfection

Neurons were infected with AMPKα shRNA lentiviral particles purchased from Santa Cruz (CA, USA) at 5d after in vitro culture according to the manufacturer’s instructions. After 48 h, the transfection efficiency was verified by WB and subsequent experiments were processed.

### Western blotting

Mice were sacrificed at different time points after reperfusion. After cardiac perfusion with PBS, the spinal column was isolated and a laminectomy was performed to expose the spinal cord. L5-L7 segments of spinal cord were carefully removed and used in subsequent experiments. Whole protein of spinal cord tissue and cells was obtained with the whole protein extraction kit (KeyGEN Biotech, Nanjing, China) according to the manufacturer’s instructions. After quantification of protein concentration with the BCA protein assay kit ((Beyotime, shanghai, China), equal amounts of protein were separated by SDS-PAGE and subsequently transferred to PVDF membranes (EMD Millipore Corp., Burlington, MA). After blocked with 5% bovine serum albumin, the membrane was incubated overnight at 4 ℃ with primary antibodies. Then the membrane was incubated with the appropriate secondary antibodies for 2 h at room temperature. Enhanced chemiluminescent reagent (Millipore) was used to visualize the immunoreactive bands. Quantification of band intensity was performed by ImageJ.

### Immunofluorescence staining assay

Tissue sections were fixed with 4% polyoxymethylene and permeabilized with 0.3% Triton X-100, then blocked with 5% BSA, and finally incubated with corresponding primary antibodies at 4 ℃ overnight. Corresponding secondary antibodies and DAPI reagent were used to treat sections the following day, and immunoactivity was visualized under a fluorescence microscope.

### Reagents and antibodies

Honokiol (HY-N0003), MK-801 (HY-15084B) and Compound C (HY-13418A) were purchased from MCE (Shanghai, China). BAPTA-AM was purchased from Thermo Fisher Scientific (Cat# B6769); cantharidin was purchased from Institute for Drug Control (Shanghai, China). All primary antibodies used in this study included: Rabbit anti-NMDAR2B (Abcam, Cambridge, UK); Fluorescein isothiocyanate (FITC) anti-SIRT3 (Biorbyt, Cambridge, UK); Rabbit anti-PGC-1α (Santa Cruz, CA, USA); Rabbit anti-AMPKα and Rabbit anti-phospho-AMPKα (Thr172) (Cell Signaling Technology, MA, USA); Rabbit anti-MAP2 (Abcam, Cambridge, UK); Mouse anti-NeuN (Abcam, Cambridge, UK); Rabbit anti- CaMKKβ and Rabbit anti-Phospho-CaMKKβ (Abcam, Cambridge, UK); Phosphatase 4 (Santa Cruz, CA, USA); Rabbit anti-GAPDH and Rabbit anti—beta Actin (Servicebio, WuHan, China).

### Statistical and data analysis

Data are shown as mean ± SD, and contain at least three independent biological replicates. One way or Two way analysis of variance (ANOVA) was used for analysis if comparisons were more than two groups, and unpaired 2 tailed Student’s t tests were used for two group comparisons. Differences between groups were considered statistically significant when p value < 0.05.

## Results

### Remote ischemic preconditioning protects mice from spinal cord ischemia–reperfusion injury

**Fig. 1:**
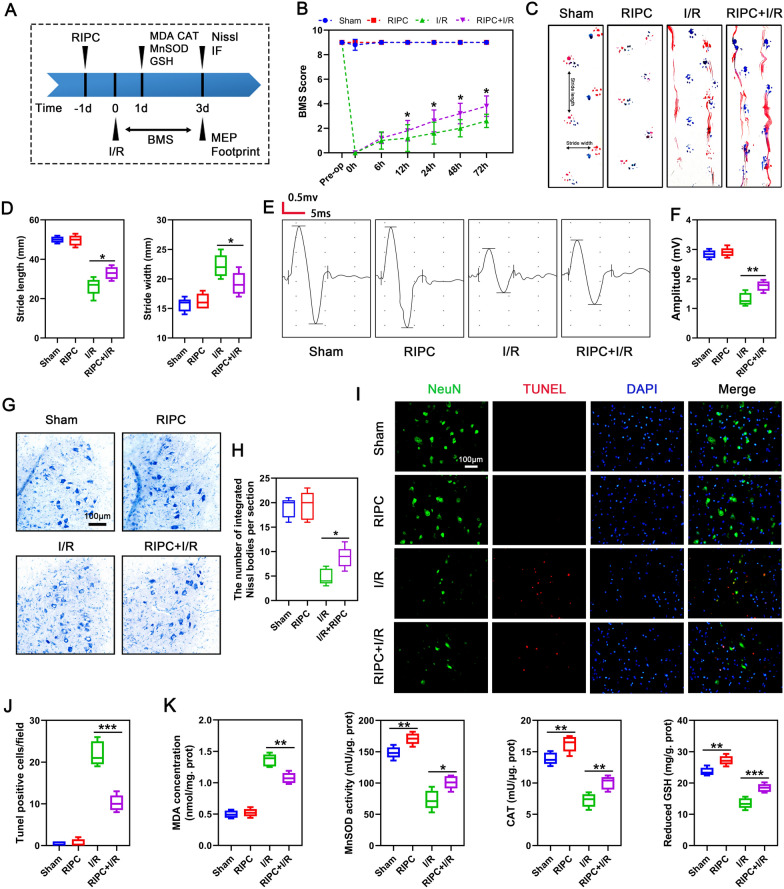
RIPC protects against SCIRI in mice. **A** Schematic diagram of the experimental design. **B** BMS scores at different time points post-injury in mice (n = 5/group). **C** Representative footprint images of mice on the third day following injury. Blue: frontpaw print; red: hindpaw print. **D** Quantification of the analysis of the footprint in each group (n = 5/group). **E** Representative images of MEP for assessing the electrophysiology of mice on the third day after injury. **F** Quantification of the peak-to-peak MEP amplitudes in each group (n = 5/group). **G** Representative images of Nissl staining of neurons in the anterior horn of the spinal cord. Scale bar = 100 μm. **H** Quantification of the number of integrated Nissl bodies per section (n = 5/group). **I** Representative images of TUNEL-positive apoptotic cells (in red) in spinal cord sections on day 3 post-injury. Neuron was stained with NeuN (in green) and nuclear stained with DAPI (in blue). Scale bar = 100 μm. **J** Quantification of the number of apoptotic cells in each group (n = 5/group). **K** MDA, MnSOD, CAT, and GSH were measured to reflected the level of oxidative stress in each group (n = 5/group). Statistical analysis: mean ± SEM, *p < 0.05, **p < 0.01, ***p < 0.001

Because the view that remote ischemic preconditioning (RIPC) protects from SCIRI is still controversia [24, 40], we first assessed if RIPC protects mice from spinal cord I/R using the strategy shown on Fig. 1A. The Basso mouse scale (BMS) analysis was performed until 3 days after ischemic. As shown in Fig. 1B, RIPC did not adversely affect the motor functions of uninjured mice, and the recovery of lower limb function within 3 days after ischemia in the RIPC + I/R group was better than in the I/R group. To assess post-ischemic hind limb balance, we performed footprint analysis on the third day after ischemia. As shown in Fig. 1C, D, mice in the RIPC + I/R group exhibited better coordination recovery, as evidenced by longer stride lengths and shorter stride width compared to the I/R group. Furthermore, we found that in the RIPC + I/R group, RIPC led to higher motor-evoked potentials (MEPs) amplitudes when compared with mice in the I/R group (Fig. 1E–F). Analysis of neuron degeneration using Nissl staining revealed that RIPC did not significantly affect Nissl body morphology and number, and that I/R was associated with weak of Nissl staining and reduced Nissl body numbers in the ventral lateral anterior horn motor neurons of the mouse lumbar spinal cord (Fig. 1G, H). This change was markedly reversed by performing RIPC before I/R. Because spinal cord I/R triggers increased neuronal apoptosis, we used TUNEL analysis to assess the rate of neuronal apoptosis. This analysis found that after I/R, the number of neurons (in green) reduced while the number of TUNEL-positive cells (red) increased, and that this change was partially reversed by RIPC (Fig. 1I, J). Redox imbalance is a key pathogenic factor in I/R injury. To determine if oxidative stress was inhibited by RIPC, we measured the levels of oxidative indexes malondialdehyde (MDA) and antioxidant indexes, including superoxide dismutase (MnSOD), catalase (CAT) and reduced-glutathione (GSH). This analysis showed that I/R reduced the activity of MnSOD and CAT, and the content of GSH in spinal cord while enhancing MDA expression (Fig. 1K). However, the above indexes were partially reversed in the RIPC + I/R group. These results indicated that RIPC-mediated inhibition of oxidative stress after spinal cord I/R is neuroprotective in mice.

### SIRT3 is essential for RIPC-mediated neuroprotection

**Fig. 2:**
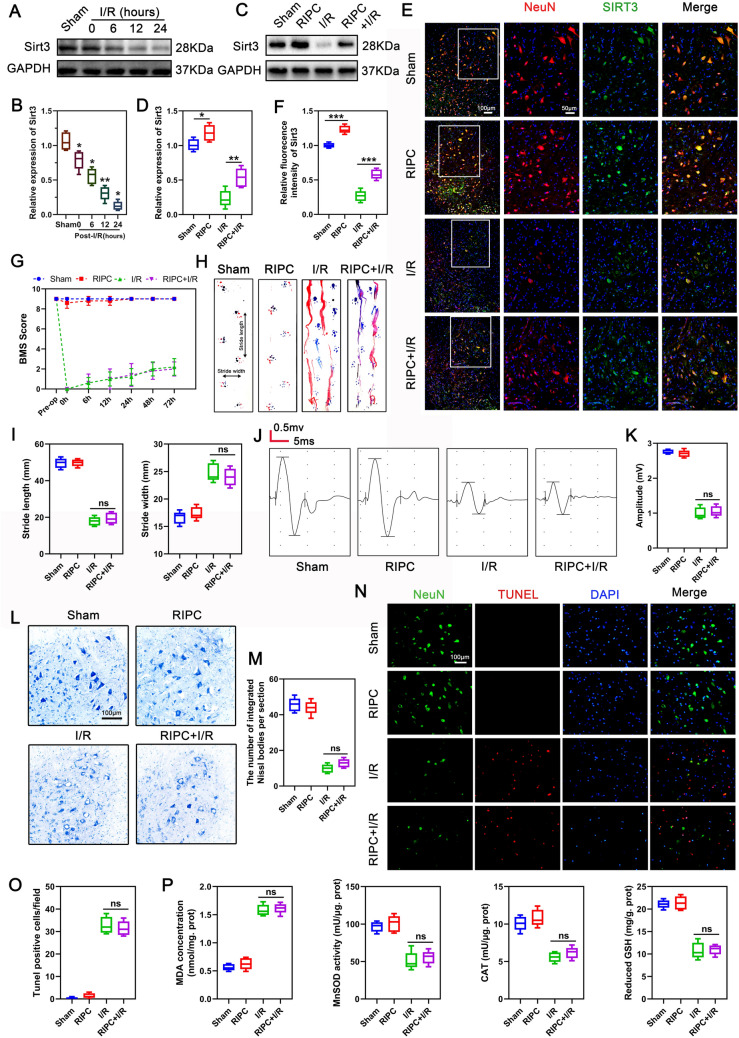
RIPC upregulates SIRT3 expression and the neuroprotective effect of RIPC is reversed in KO mice. **A**, **B** Western blot was used to detect the expression of SIRT3 at different time points after spinal cord I/R. Relative expression of SIRT3 was normalized to the level of control. N = 5/group, *p < 0.05, **p < 0.01, compared to the former timepoint. **C**, **D** Western blot was used to detect the expression of SIRT3 in different groups. Relative expression of SIRT3 was normalized to the level of the sham group (n = 5/group). **E**, **F** Immunofluorescence was used to detect the expression of SIRT3 (green) in neurons (red) in different groups. Nuclear was stained with DAPI (in blue). Scale bar = 100 μm. Relative fluorescence intensity was normalized to the level of the sham group (n = 5/group). **G** The KO mice were functionally scored up to 3d postinjury using BMS (n = 5/group). **H**, **I** Representative footprint images of KO mice on the third-day post-injury and quantification of the stride length and width. Blue: frontpaw print; red: hindpaw print (n = 5/group). **J**, **K** MEP analysis was used for electrophysiological assessment at day3 postinjury and quantification of the peak-to-peak MEP amplitudes in KO mice (n = 5/group). **L**, **M** Representative images of Nissl staining of neurons in the anterior horn of the spinal cord and quantification of the number of integrated Nissl bodies per section from KO mice (n = 5/group). Scale bar = 100 μm. **N**, **O** Representative images of TUNEL-positive apoptotic cells (in red) in spinal cord sections and quantification of the number of apoptotic cells in each group from KO mice at day 3 postinjury (n = 5/group). Neuron was stained with NeuN (in green) and Nuclear stained with DAPI (in blue). Scale bar = 100 μm. **P** MnSOD, GSH, CAT, and MDA were measured to reflect the level of oxidative stress in each group of KO mice (n = 5/group). Statistical analysis: mean ± SEM, *p < 0.05, **p < 0.01, ***p < 0.001

To investigate the role of SIRT3 in RIPC-mediated neuroprotection, we first examined SIRT3 expression at various timepoints after SCIRI. Western blot revealed that SIRT3 levels fell significantly 6 h after reperfusion and remained low for 24 h (Fig. 2A, B), indicating that SIRT3 downregulation may contribute to the deterioration of neurological function in mice after SCIRI. Next, we investigated whether the neuroprotective effects of RIPC was mediated by SIRT3, western blotting results showed that RIPC enhanced SIRT3 expression and partially rescued SIRT3 downregulation caused by I/R (Fig. 2C, D). This conclusion was further confirmed by immunofluorescence staining of SIRT3 in neurons (Fig. 2E, F). To further determine if SIRT3 was necessary for RIPC-mediated neuroprotection, we generated Sirt3-KO mice as previously reported [41] and subjected them to RIPC and I/R injury. Genotyping of KO mice was confirmed using DNA that was isolated from tail clips (Additional file 1: Fig. S1). Analysis of mouse neurological recovery using BMS scores, footprint analysis, and MEPs revealed that, when compared with WT mice (Fig. 1B–F), RIPC did not enhance functional recovery in KO mice (Fig. 2G–K). Moreover, Nissl staining showed that RIPC did not reverse I/R-induced loss of Nissl bodies and morphological changes in KO mice (Fig. 2L, M). The number of TUNEL-positive cells in RIPC + I/R group KO mice was similar to that observed in I/R group (Fig. 2N, O). These results indicated that SIRT3 was essential for RIPC-mediated neuroprotection. To determine if SIRT3 contributes to RIPC-mediated neuroprotection by regulating the activity of antioxidant enzymes, we assessed the activity of SOD, and CAT, as well as GSH content. Results showed that RIPC-induced activation of SOD, CAT, and GSH in WT mice were markedly suppressed in KO mice (Fig. 2P). Moreover, I/R induced a more significant increase in MDA content in KO mice when compared to WT mice, and this effect was not significantly ameliorated by RIPC. Together, these results indicated that SIRT3 plays a key role in RIPC-mediated tolerance to I/R and resistance to oxidation in vivo.

### RIPC regulates SIRT3 expression via NMDAR

**Fig. 3:**
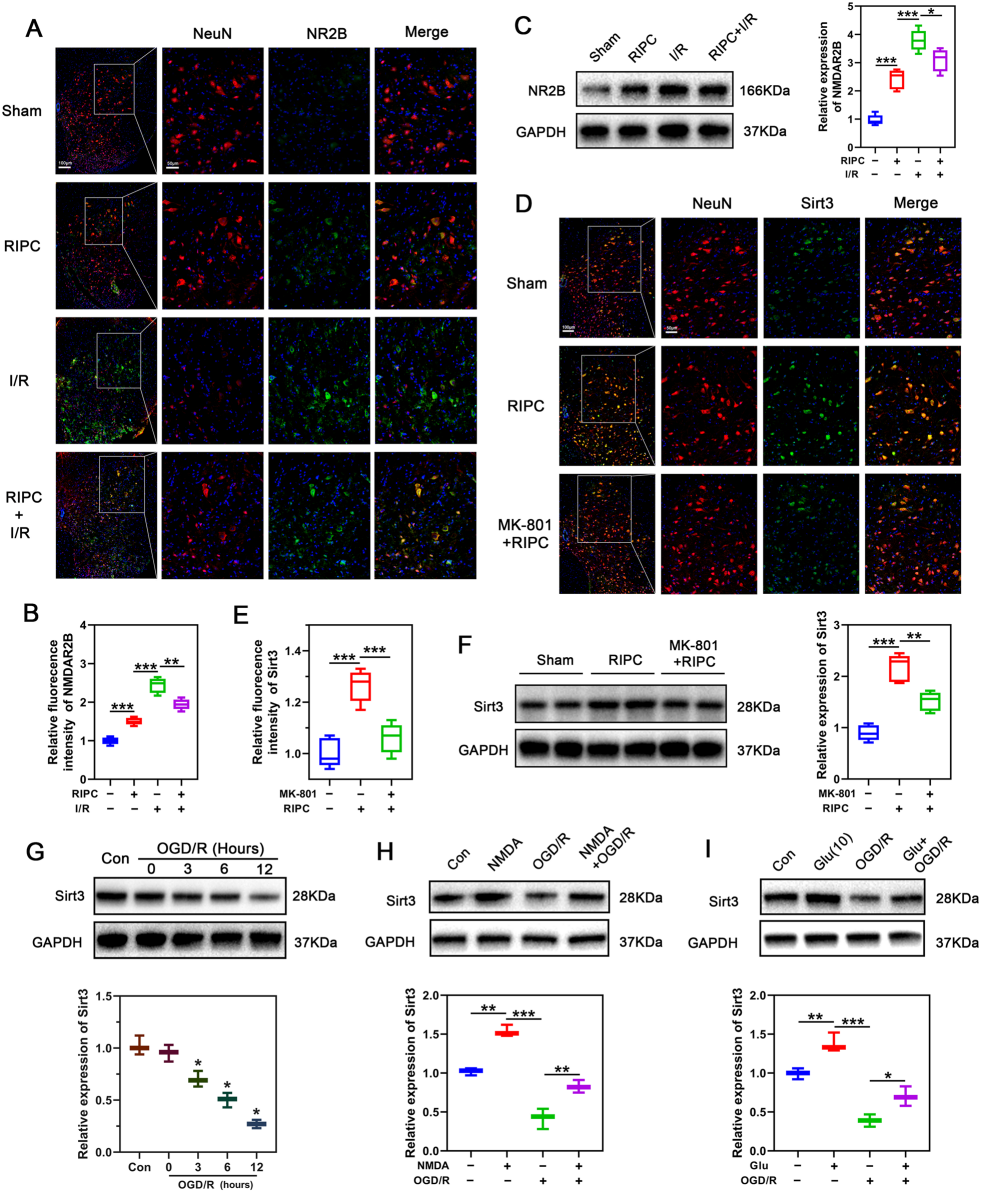
RIPC fails to upregulate SIRT3 after NMDAR inhibition. **A**,** B** Immunofluorescence was used to detect the expression of NMDAR2B (in green) in neurons (in red) in different groups (n = 5/group). Nuclear was stained with DAPI (in blue). Scale bar = 100 μm. **C** Western blotting was used to detect the expression of NMDAR2B in different groups (n = 5/group). **D**, **E** The expression of SIRT3 (in green) in the spinal cord neurons (in red) was detected by immunofluorescence after administration of NMDR2B inhibitor MK-801 (n = 5/group)**.** Scale bar = 100 μm. **F** The expression of SIRT3 in the spinal cord was detected by Western blotting after administration of MK-801 (n = 5/group)**. G** Western blotting was used to detect the expression of SIRT3 at different time points after OGD/R (n = 5/group). **H** The expression of SIRT3 in neurons was detected by Western blotting after different treatment conditions and NMDA partially reversed the down-regulation of SIRT3 in neurons after OGD/R (n = 3/group). **I** The expression of SIRT3 in neurons was detected by Western blotting after different treatment conditions and sublethal glutamate partially reversed the down-regulation of SIRT3 in neurons after OGD/R (n = 5/group). Statistical analysis: mean ± SEM, *p < 0.05, **p < 0.01, ***p < 0.001

Although we found that RICP protects neurons from SCIRI by modulating SIRT3, it is not clear how RIPC induces SIRT3 expression. Previous finding have shown that RIPC enhances glutamic levels in the spinal ventral horn and sub-lethally activates N-methyl-D-aspartate receptor (NMDAR) [34]. Several studies have shown that NMDAR is involved in the protective effects of IPC [42, 43]. However, it is not clear if NMDAR participates in RIPC-mediated neuroprotection by modulating SIRT3. To determine the relationship between NMDAR and SIRT3, immunofluorescence was performed to assess NMDAR expression in spinal cord neurons. As shown in Fig. 3A, B, NMDAR levels were elevated by RIPC and further enhanced after I/R. However, performing RIPC before I/R attenuated SCIRI-induced NMDAR overactivation (Fig. 3A, B). Similar effects were observed through western blotting (Fig. 3C). Next, to inhibit the NMDA receptor, we intravenously treated preconditioned mice with NMDA receptor inhibitor, dizocilpine (MK-801, 1 mg/kg), 60 min before remote ischemic preconditioning and evaluated SIRT3 expression. Immunofluorescence and western blot revealed that MK-801 suppressed RIPC-induced SIRT3 upregulation (Fig. 3D–F), indicating that RIPC may regulate SIRT3 expression through glutamate and the glutamate receptor, NMDAR.

### SIRT3 deficiency exacerbates OGDR-induced neuronal injury and suppresses NMDAR agonists-mediated protection in vitro

**Fig. 4:**
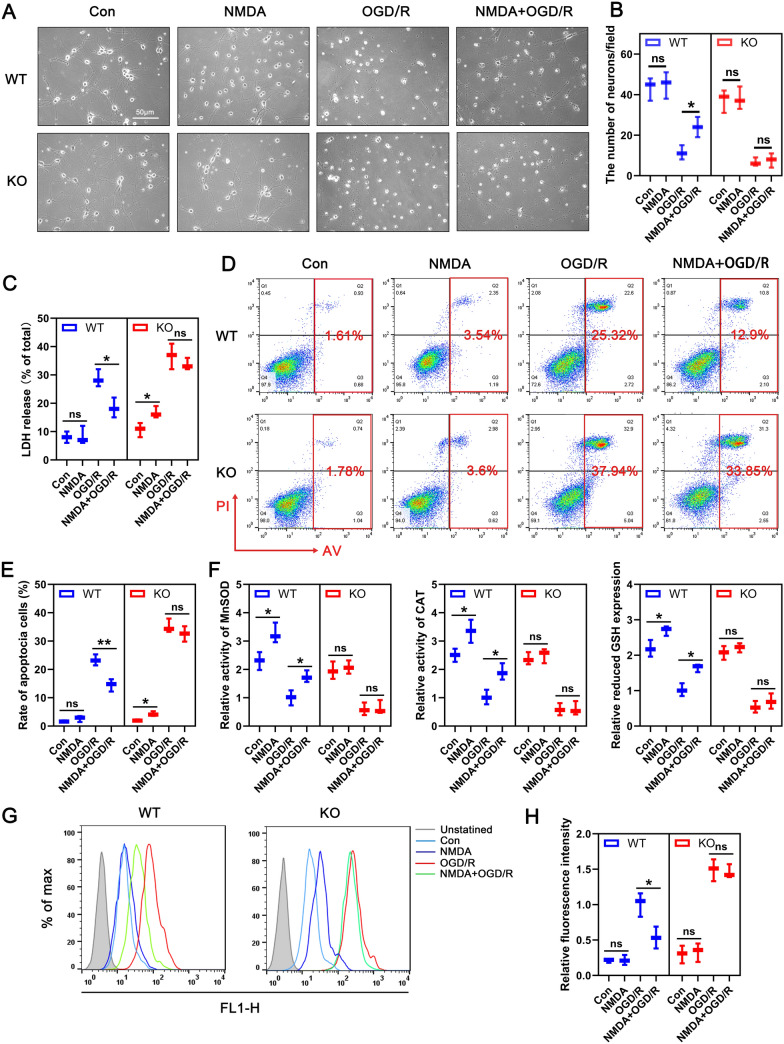
NMDA exerts a neuroprotective effect similar to that of RIPC in vitro. **A** Representative brightfield image showing morphologic changes of primary neurons in WT or KO*-*derived neurons after NMDA pretreatment. Scale bar = 50 μm. **B** Quantification of surviving neurons in figure A (n = 3/group). **C** The percentage of released LDH from OGD/R-treated KO or WT- derived neurons was assessed to determine the neuronal injury in the presence or absence of NMDA (n = 3/group). **D** Flow cytometry was used to detect the apoptosis of WT or KO-derived neurons pretreated with or without NMDA by PI/Annexin V double labeling. **E** Quantification of apoptotic cells (Annexin V-positive) in panel C (n = 3/group). **F** Relative activity of MnSOD, CAT, and GSH content was detected in neurons. Relative activity or content was normalized to the level of WT-derived neurons treated with OGD/R (n = 3/group). **G** Analysis of ROS production by flow cytometry in KO (left panel) and WT (right panel) derived neurons pretreated with or without NMDA. **H** Quantification of ROS production in neurons of indicated groups (n = 3/group). Relative fluorescence intensity was normalized to the level of WT neurons that were treated with OGD/R. Statistical analysis: mean ± SEM, *p < 0.05, **p < 0.01, ***p < 0.001

To further investigate the relationship between NMDAR and SIRT3, we extracted primary cortical neurons from WT and KO embryonic mice, and performed oxygen–glucose deprivation and reoxyglucose (OGD/R) to simulate ischemia–reperfusion injury. The purity of the neurons was identified by co-staining MAP2 and NeuN, and neurons with purity greater than 90% were used in subsequent experiments (Additional file 1: Fig. S2). Next, we examined SIRT3 levels in response to OGD/R. Similar to the in vivo results, neuronal SIRT3 expression reduced with increasing duration of OGD/R treatment (Fig. 3G). Neuronal Stimulation using the NMDAR agonist, NMDA (100 μm) or sublethal glutamate (10 μm) for 6 h markedly elevated SIRT3 expression and reversed OGD/R-induced SIRT3 downregulation (Fig. 3I–H). These results confirmed that NMDAR may control neuronal ischemic tolerance by modulating SIRT3 expression. To determine if NMDA protects neurons from OGD/R in a SIRT3-dependent manner, neurons obtained from WT and KO embryonic mice were preconditioned respectively with NMDA for 6 h followed by OGD/R. As shown in Fig. 4A, B, bright field images revealed that NMDA treatment alone had no significant effect on the morphology and number of WT or KO mice derived neurons. However, KO-derived neurons were more susceptible to OGD/R stimulation and the resulting damage could not be reversed by NMDA preconditioning. In contrast, NMDA pretreatment improved the morphology and abundance of WT*-*derived neurons after OGD/R. Similarly, SIRT3 deficiency led to more toxic LDH release from neurons, which could not be reversed by NMDA (Fig. 4C). Flow cytometry showed that NMDA pretreatment suppressed apoptosis of WT*-*derived neurons but not in KO*-*derived neurons (Fig. 4D, E). In addition, NMDA pretreatment enhanced the activities of the antioxidant enzymes MnSOD and CAT and increased the level of GSH in WT*-*derived, but had no similar effect on KO*-*derived neurons (Fig. 4F). Flow cytometry was used to detected ROS generation. The results showed that pretreatment with NMDA suppressed ROS generation in WT*-*derived neurons after OGD/R, but had no significant effect on ROS production in KO*-*derived neurons (Fig. 4G, H). Together, these results indicate that SIRT3 plays a key role in NMDAR-mediated ischemic tolerance in vitro.

### NMDAR regulates SIRT3 expression via the AMPK/PGC-1α signaling pathway

**Fig. 5:**
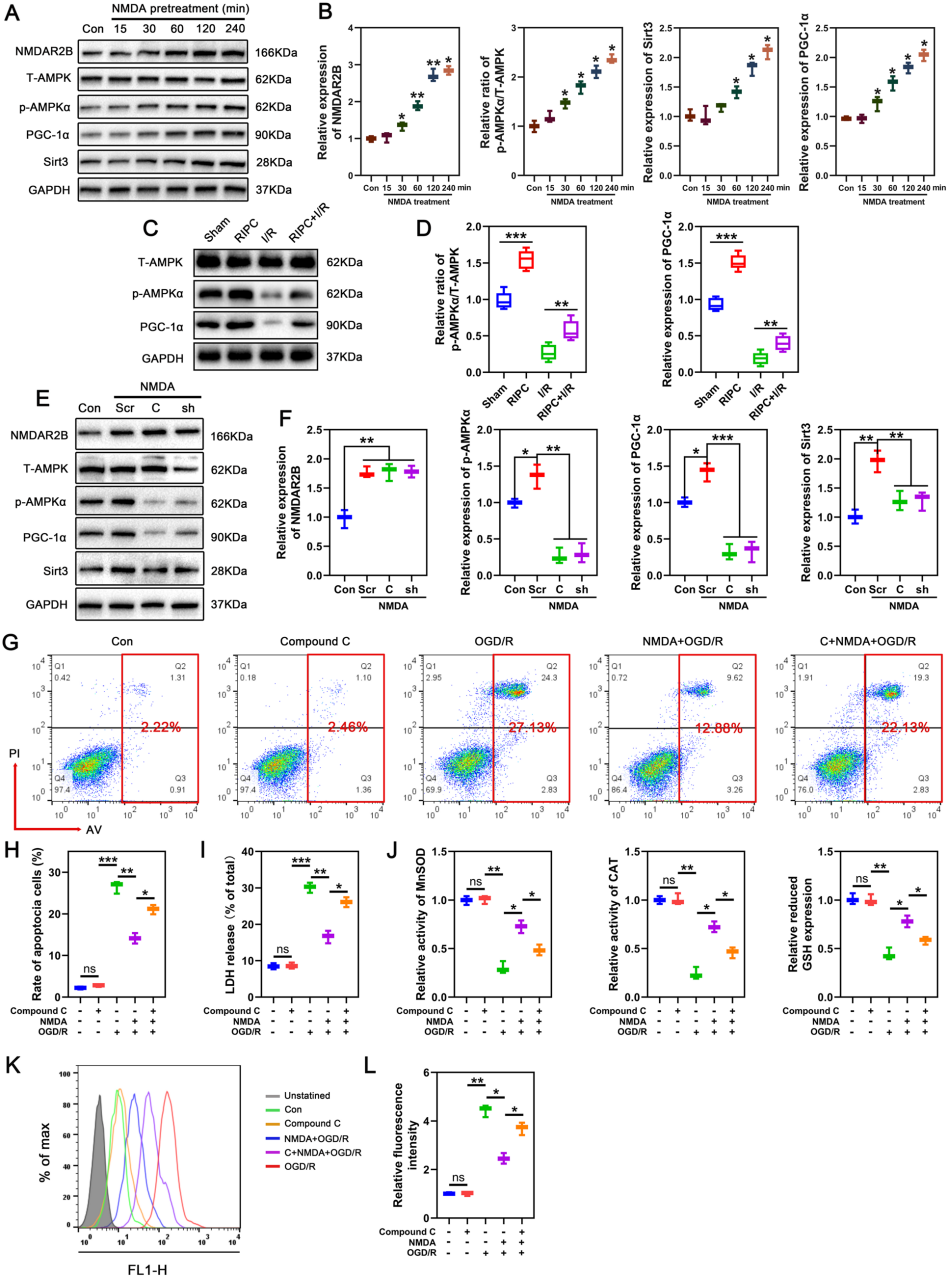
SIRT3 expression is regulated by the classical AMPK/PGC-1α signaling pathway and inhibition of AMPK attenuates the NMDA-induced neuroprotective effect. **A**, **B** Altered protein expression of NMDAR2B, T-AMPK, p-AMPK, PGC-1α and SIRT3 in neurons treated with NMDA was detected by Western blotting at different time points. Relative expression was normalized to the level of the control (n = 3/group). **C**, **D** Western blotting was used to detect the expression of T-AMPK, p-AMPK, and PGC-1α after sham or I/R treatment with or without RIPC. Relative expression was normalized to the level of the sham group (n = 5/group). **E**, **F** In response to NMDA, altered protein expression levels of p-AMPK, PGC-1α and SIRT3 in neurons when blocking AMPK using AMPK shRNA and compound C (an AMPK inhibitor) were detected using Western blotting. Relative expression was normalized to the level of control (n = 3/group). **G** Flow cytometry was used to detect the effect of compound C pretreatment on apoptosis of NMDA-OGD/R treated neurons (n = 3/group). **H** LDH was detected to assess neuronal damage after pretreatment with compound C in NMDA-OGD/R treated neurons (n = 3/group). **I** The relative activity of MnSOD, CAT, and GSH content was measured after pretreatment with compound C in NMDA-OGD/R treated neurons (n = 3/group). **J**, **K** The production of ROS was measured by flow cytometry after pretreatment with compound C in NMDA-OGD/R treated neurons (n = 3/group). Statistical analysis: mean ± SEM, *p < 0.05, **p < 0.01, ***p < 0.001

To determine the mechanism by which NMDAR modulates SIRT3 expression, we examined the AMPK/PGC-1α signaling pathway, which is reported to act upstream mediator of SIRT3. As shown in Fig. 5A, B, NMDA increased AMPK phosphorylation and PGC-1α as well as SIRT3 expression in a time-dependently manner in neurons. In vivo, RIPC also upregulated the levels of p‐AMPK and PGC-1α in the spinal cord, and reversed p‐AMPK and PGC-1α downregulation induced by I/R (Fig. 5C, D). To further confirm that AMPK is the upstream mediator of RIPC regulating SIRT3 expression, an AMPK inhibitor, compound C and shRNA were used to treated neurons. As shown in Fig. 5E, F, NMDA-induced upregulation of SIRT3 and PGC-1α was significantly suppressed after AMPK inhibition. To identified if AMPK is also involved in SIRT3-dependent neuronal survival and mitochondrial homeostasis, we also assessed the effects of NMDA on neuronal apoptosis and oxidative stress after AMPK inhibition. As shown in Fig. 5G–I, compound C did not affect neuronal viability under normal conditions, but significantly reversed the inhibitory effect of NMDA on OGD/R-induced apoptosis and LDH release. The activity of antioxidant enzymes and the content of GSH and ROS were also measured after AMPK inhibition. As shown in Fig. 5J, AMPK inhibition using compound C after NMDA + OGD/R treatment suppressed the activities of MnSOD and CAT as well as the levels of GSH, while enhancing ROS production (Fig. 5K, L). Together, these results suggest that NMDAR/AMPK/PGC-1α signaling promotes mitochondrial homeostasis and neuronal survival by upregulating SIRT3.

### Biphasic effects of different Ca^2+^ concentrations on AMPK activation

**Fig. 6:**
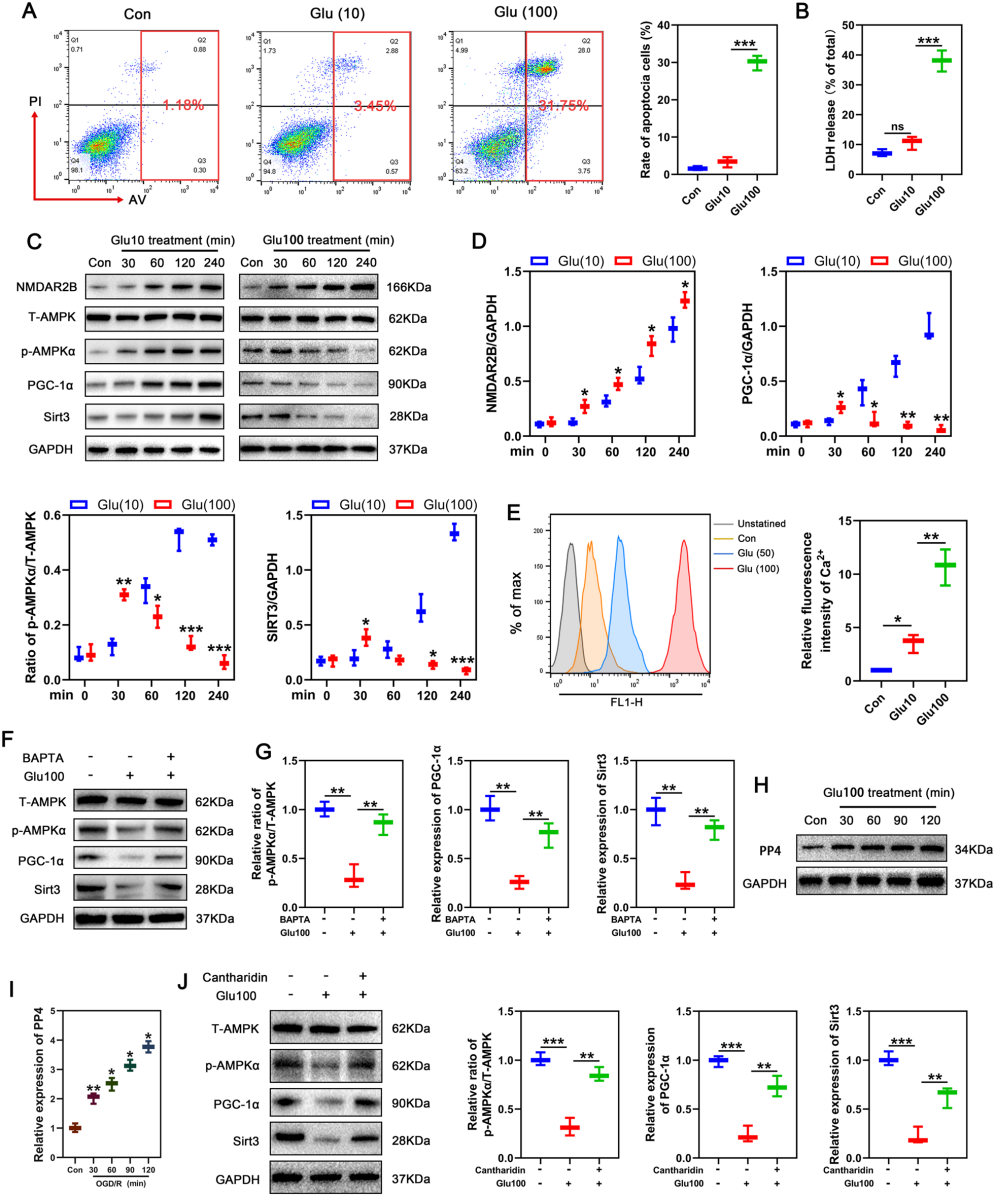
Toxic concentrations of glutamate inhibits AMPK activation through PP4 activation. **A** Flow cytometry was used to detect the apoptosis of neurons stimulated with different concentrations of glutamate. Apoptotic neurons were defined as Annexin V-positive cells (n = 3/group). **B** LDH was detected to assess neuronal damage after stimulation with different concentrations of glutamate (n = 3/group). **C**, **D** Altered protein expression levels of NMDAR2B, T-AMPK, p-AMPK, PGC-1α and SIRT3 in neurons treated with different concentrations of glutamate were detected by Western blotting at different time points (n = 3/group). **E** Flow cytometry was used to detect intracellular Ca^2+^ concentration in neurons stimulated with different concentrations of glutamate. Relative intensity was normalized to the level of control (n = 3/group). **F**, **G** Western blotting was used to detect the expression of T-AMPK, p-AMPK, PGC-1α and SIRT3 after treatment with intracellular calcium chelator BAPTA in 100Glu-treated neurons. Relative expression was normalized to the level of control (n = 3/group). **H**, **I** The expression of protein phosphatase-4 was detected using western blotting in neurons after treatment with 100Glu at different time points. Relative expression was normalized to the level of control. * p < 0.05, **p < 0.01, compared to the former timepoint (n = 3/group). **J** Western blotting was used to detect the expression of T-AMPK, p-AMPK, PGC-1α and SIRT3 after treatment with the PP4 inhibitor cantharidin in 100Glu-treated neurons. Relative expression was normalized to the level of control (n = 3/group). Statistical analysis: mean ± SEM, *p < 0.05, **p < 0.01, ***p < 0.001

As mentioned above, RIPC increased SIRT3 expression through activation of NMDAR. However, after I/R, NMDAR was further activated, but the level of SIRT3 was no longer increased. This observation suggested a biphasic effect of NMDAR on SIRT3 regulation. In vitro*,* neurons were treated with sublethal (10 μM) and lethal concentrations (100 μM) of glutamate to mimic NMDAR activation by RIPC and I/R in vivo respectively. Neuronal apoptosis and LDH release were detected. The results showed that sublethal glutamate had no effect on neuronal viability, but lethal glutamate resulted in significant neuronal apoptosis and LDH release (Fig. 6A, B). Western blot revealed that sublethal concentrations activated the AMPK/PGC-1/SIRT3 pathway in a time-dependent manner, while lethal glutamate levels markedly inhibited it (Fig. 6C, D). This suggests that various NMDAR activation levels may determine neuronal fate by modulating the AMPK/SIRT3 signaling.

The activation of NMDAR by ligands triggers inward calcium flow. It has been reported that transient Ca^2+^ level elevation contributed to AMPK activation, while sustained high Ca^2+^ levels inhibited AMPK activation. Therefore, we speculated that the above difference may be due to different intracellular Ca^2+^ levels in response to distinct stimuli. To test this possibility, we used Fluo-4, a fluorescent probe, to assess changes in intracellular Ca^2+^ levels under various stimuli using flow cytometry. As shown in Fig. 6E, 100 μM glutamate caused a markedly higher inward flow of intracellular calcium when compared with 10 μM glutamate. To elucidate the relationship between intracellular Ca^2+^ concentration and AMPK activation, neurons were treated with the intracellular calcium chelator BAPTA-AM (10 μM) [44] before lethal glutamate administration. Western blot revealed that pretreatment with BAPTA-AM restored AMPK activation as well as the expression of PGC-1α and SIRT3 (Fig. 6F). This observation highlighted the biphasic nature of Ca^2+^ concentration in AMPK phosphorylation. Calcium/calmodulin-dependent protein kinase β (CaMKKβ) is a serine/threonine-protein kinase belonging to the Ca^2+^/calmodulin-dependent protein kinase subfamily [45], and AMPK-α is a key CaMKKβ target [46]. Thus, we hypothesized that toxic concentrations of glutamate inhibited AMPK activation by inhibiting CaMKKβ autophosphorylation. However, western blot analysis revealed that treating neurons with toxic glutamate concentrations (100 μM) did not inhibit CaMKKβ activation but instead, enhanced CaMKKβ phosphorylation when compared with 10Glu glutamate (Additional file 1: Fig. S3). Indicating that AMPK activity was suppressed via CaMKKβ-independent mechanism. Because Ca^2+^ levels may enhance AMPK dephosphorylation by protein phosphatase-4 (PP4) [44], we evaluated neuronal PP4 protein levels after treatment with lethal glutamate concentration (100 μM). Western blot analysis revealed that the lethal glutamate concentration elevated neuronal PP4 protein levels (Fig. 6H, I), indicating a potential link between PP4 expression levels and AMPK activity. Next, we pretreated neurons with the PP4 inhibitor, cantharidin (50 μM), before stimulating them with the lethal glutamate concentration. Western blot showed that cantharidin restored AMPK phosphorylation as well as the expression of PGC-1α and SIRT3 (Fig. 6J), which is consistent with the calcium chelators (Fig. 6F). These findings indicate that NMDAR regulates AMPK activation via inward Ca^2+^ flow and that transient Ca^2+^ elevation contributes to AMPK activation, while sustained high Ca^2+^ levels inhibit AMPK activation by upregulating PP4 expression in neurons.

### Honokiol, a SIRT3 agonist, protects neurons from OGD/R-induced oxidative stress damage

**Fig. 7:**
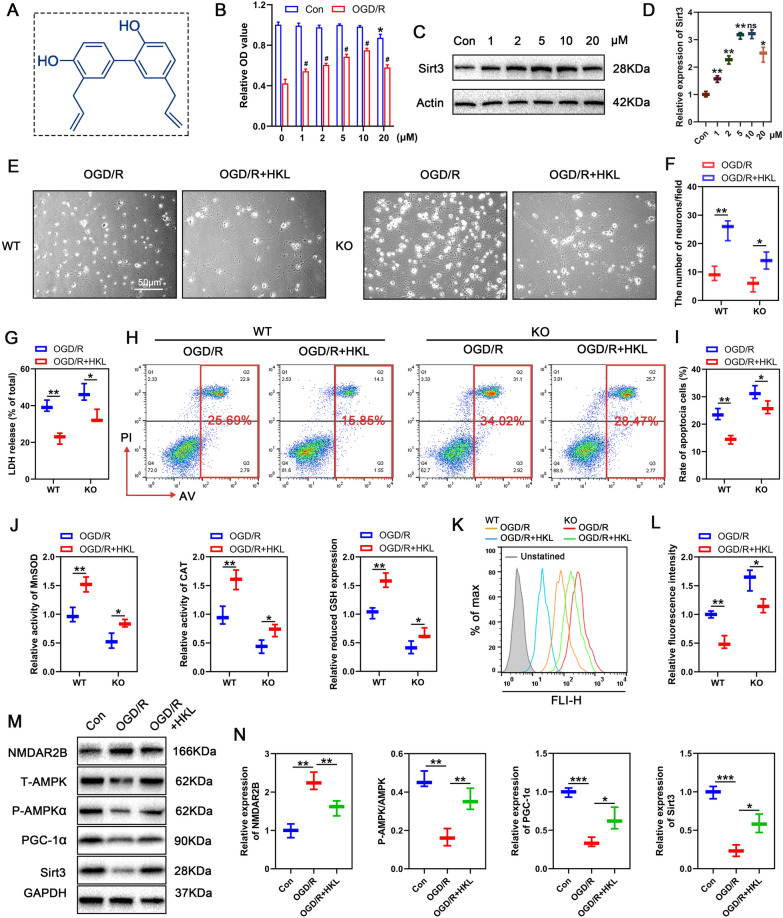
HKL attenuates OGD/R-induced damage by preventing neuronal apoptosis and oxidative stress via the NMDAR/AMPK/PGC-1α/SIRT3 pathway in neurons. **A** The chemical structure of HKL. **B** The CCK8 kit was used to detect the viability of neurons that were treated with different concentrations of HKL with or without OGD/R (n = 6/group). * p < 0.05, compared to the former group. **C**, **D** Immunoblot images of SIRT3 in neurons treated with different concentrations of HKL. Relative expression was normalized to the level of the control treatment (n = 3/group). *p < 0.05, **p < 0.01, compared to the former group. **E** Representative brightfield images showing morphologic changes of OGD/R-treated primary neurons in the presence or absence of HKL. Scale bar = 50 μm. **F** Quantification of surviving neurons in figure E (n = 3/group). **G** The percentage of released LDH from OGD/R-treated KO or WT-derived neurons was assessed to determine neuronal injury in the presence or absence of HKL (n = 3/group). **H** Representative scatter plots of apoptotic neurons induced by OGD/R through PI/Annexin V double labeling in the presence or absence of HKL. **I** Quantification of apoptotic neurons (Annexin V-positive; n = 3/group) in panel **G**. **J** Relative activity of MnSOD, CAT, and GSH content in KO or WT-derived neurons after OGD/R treatment with or without HKL (n = 3/group). **K** Flow cytometry analysis of ROS production in KO or WT-derived neurons after OGD/R treatment in the presence or absence of HKL. **L** Quantification of ROS production in neurons of indicated groups (n = 3/group). Normalized to the level of WT neurons that were treated with OGD/R. **M**, **N** Western blotting was used to determine the effect of HKL on the expression of NMDAR2B, T-AMPK, p-AMPK, PGC-1α and SIRT3 in neurons that were subjected to OGD/R. Relative expression was normalized to the level of the control treatment (n = 3/group). Statistical analysis: mean ± SEM, *p < 0.05, **p < 0.01, ***p < 0.001

Despite the considerable potential of RIPC in resistance to SCIRI, we face the challenge of what else can be done after SCIRI. Enhancing neuronal SIRT3 expression before or after injury in order to improve resistance to external stress is a promising direction. Honokiol (HKL, Fig. 7A), a biphenolic compound obtained from the bark of magnolia trees, has been reported to have antioxidative and neuroprotective properties. Moreover, HKL promotes SIRT3 activity by enhancing SIRT3 expression as well as directly binding to SIRT3 and enhancing its deacetylase activity [47, 48]. Thus, HKL may have significant therapeutic potential against SCIRI. To test this possibility, we first assessed the effects of different HKL concentrations on neuronal viability and SIRT3 expression. CCK8 analysis showed that neurons treated with HKL at 10 μM exhibited maximum viability in response to OGD/R, and had no obvious toxic effect on normal neurons (Fig. 7B). Consistently, western blot also revealed that neuronal SIRT3 expression was highest upon treatment with 10 μM HKL in vitro (Fig. 7C, D). Therefore, 10 μM HKL was used in subsequent experiments. Next, we performed oxy-glucose deprivation and then added HKL to the normal neuronal media after re-oxy-glucose onset and maintained for 12 h. Brightfield microscopic examination revealed that HKL partially rescued OGDR-induced neuronal morphological changes and loss (Fig. 7E, F). HKL-mediated resistance to OGD/R was further confirmed by measuring the level of LDH release into the neuronal medium (Fig. 7G). Flow cytometry revealed that HKL suppressed OGD/R-induced neuronal apoptosis (Fig. 7H, I). Notably, we observed that the neuroprotective effects of HKL were markedly attenuated in *Sirt3*^−/−^*-*derived neurons (Fig. 7E–I). Moreover, HKL enhanced the activity of the antioxidant enzymes MnSOD and CAT, increased the GSH level (Fig. 7I), and inhibited OGD/R-induced mitochondrial ROS production in the presence of SIRT3 (Fig. 7J–L). However, this antioxidant effect of HKL was markedly attenuated in *Sirt3*^−/−^*-*derived neurons (Fig. 7J–L). Importantly, we found that in OGD/R treated neurons, HKL upregulated SIRT3 expression by activating AMPK-PGC-1α signaling and suppressed NMDAR overaction (Fig. 7M, N). These results indicate that HKL protects neurons from OGD/R-induced oxidative stress by at least in part, modulating NMDAR/AMPK/PGC-1α/SIRT3 signaling.

### HKL protects mice from SCIRI partially depend on SIRT3

After confirming the inhibitory effect of HKL on neuronal apoptosis and oxidative stress in vitro, we then tested the effect of HKL on neurological function recovery in SCIRI mice. HKL treatment (0.2 mg/kg/day, intraperitoneally) [48] was started on the day after surgery and maintained throughout the study. Since HKL is not a SIRT3 specific inhibitor, we also examined the effect of HKL on motor function recovery in KO mice. BMS score, footprint analysis and MEPs results suggested that HKL could effectively improve the recovery of lower limb motor function in SCIRI mice. However, in KO mice, although HKL also partially restored the neurological function of SCIRI mice, this neuroprotective effect was significantly weakened compared with WT mice (Additional file 1: Fig. S4A–E). Further studies suggested that HKL increased the number of Nissl bodies in the spinal anterior horn of SCIRI mice (Additional file 1: Fig. S 4F, G), reduced TUNEL positive cells (Additional file 1: Fig. S4H, I), and improved the reoxidation-reduction imbalance of spinal tissue (Additional file 1: Fig. S4J), but the above effects were also significantly attenuated in KO mice (Additional file 1: Fig. S4F–J). These results suggest that HKL protects mice against SCIRI, and the neuroprotective effect is partly dependent on the expression of SIRT3.

### RIPC-HKL combination exhibits better efficacy against SCIRI

**Fig. 8:**
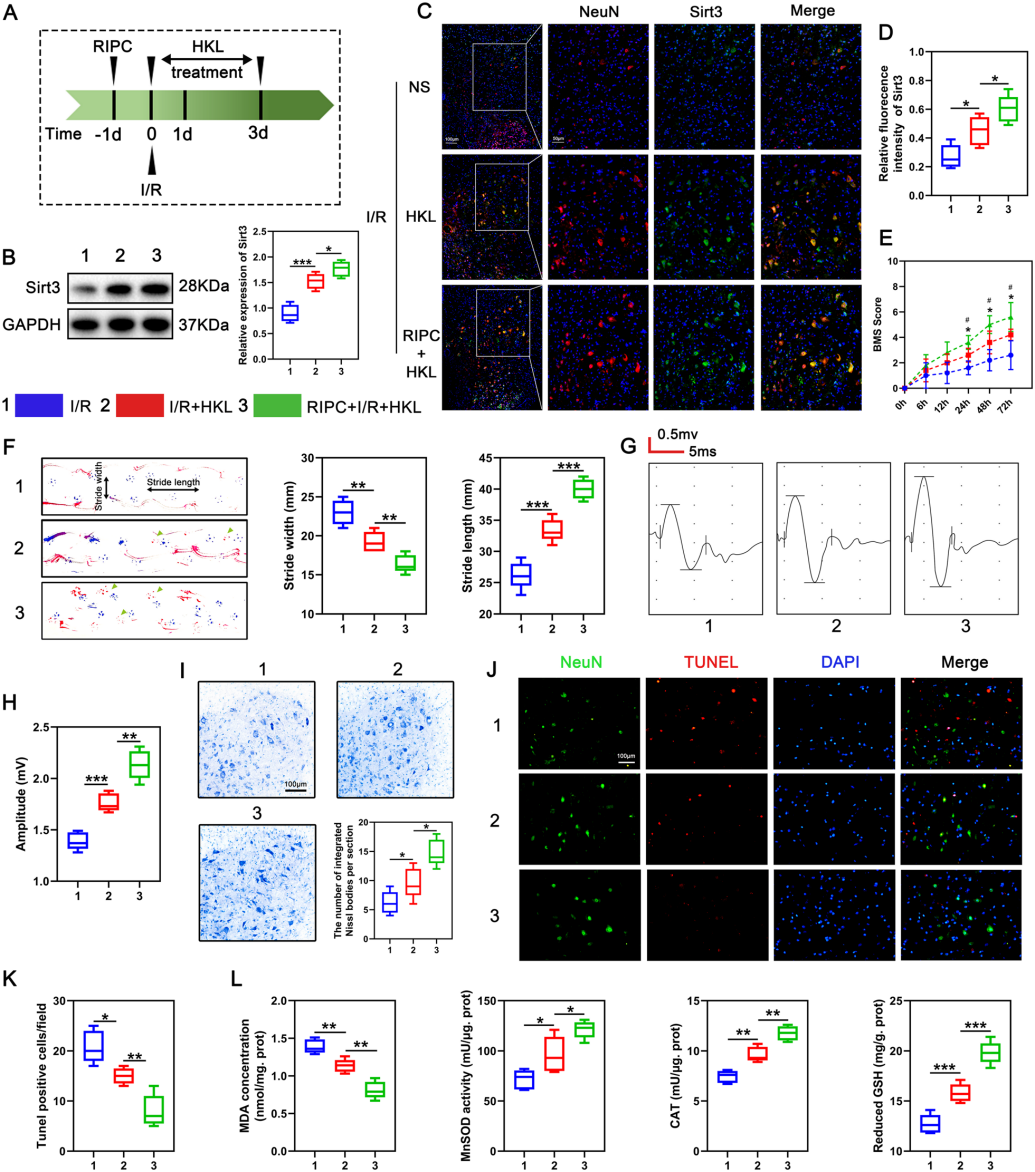
RIPC combined with HKL achieves better efficacy in mice with SCIRI. **A** Schematic diagram of experimental design. **B** Representative immunoblot images showing the effect of HKL and combined therapy on the expression of SIRT3 after spinal cord I/R (n = 5/group). **C**, **D** Immunofluorescence was used to detect the effect of HKL and combined therapy on the expression of SIRT3 (in green) in neurons (in red) after spinal cord I/R (n = 5/group). Nuclear was stained with DAPI (in blue). Scale bar = 100 μm. **E** The mice were functionally scored up to 3d postinjury using the BMS after treatment with HKL or combined therapy (n = 5/group). * p < 0.05, HKL treatment group compared to I/R injury group; # p < 0.05, Combination treatment group compared to HKL treatment group. **F** Representative footprint images from mice on the third day after injury after treatment with HKL or combined therapy and quantification of the stride length and width (n = 5/group). Blue: frontpaw print; red: hindpaw print. **G**, **H** MEP analysis was used as an electrophysiological assessment in mice treated with HKL or combined therapy on day 3 and quantification of the peak-to-peak MEP amplitudes (n = 5/group). **I** Representative images of Nissl bodies in the anterior horn of the spinal cord and quantification of the number of integrated Nissl bodies per section (n = 5/group). Scale bar = 100 μm. **J** Representative images of TUNEL-positive apoptotic cells (in red) in spinal cord sections on day 3 after I/R injury and quantification of the number of apoptotic cells in each group (n = 5/group). Neuron was stained with NeuN (in green) and Nuclear was stained with DAPI (in blue). Scale bar = 100 μm. **K** MnSOD, GSH, CAT, and MDA were measured on day 3 after injury to reflect the level of oxidative stress (n = 5/group). Statistical analysis: mean ± SEM, *p < 0.05, **p < 0.01, ***p < 0.001

Based on our findings that HKL is neuroprotective against SCIRI, and that RIPC and HKL protect from SCIRI by independently modulating SIRT3 expression, we assessed if combining HKL and RIPC can enhance their individual protective effects against SCIRI. Therefore, preischemic RIPC and sustained HKL administration after ischemia were combined to treat SCIRI and named combined therapy (Fig. 8A). To test if the effects of two regimens were additive, we compared the efficacy of the combined therapy with that of HKL alone. We first compared the effect of HKL and that of combined therapy on SIRT3 expression in the spinal cord. Western blot results showed that HKL increased SIRT3 level compared with I/R group, and this effect was more obvious when combined with RIPC (Fig. 8B). Similar results were obtained by immunofluorescence (Fig. 8C, D). These results indicated that combining RIPC with HKL was more effective than HKL alone in rescuing SIRT3 expression after SCIRI. Next, we investigated if the upregulation of SIRT3 by HKL alone or the combined therapy restored mouse neurological function. BMS revealed that mice motor function started to improve 24 h after administration with HKL and was further improved by combining HKL with RIPC (Fig. 8E). Moreover, footprint analysis on day 3 after SCIRI revealed that the hindlimb coordination of mice treated with combination therapy were better than that of mice treated with HKL alone (Fig. 8F). The amplitude of MEPs in mice treated with combination therapy was also higher than that treated with HKL alone (Fig. 8G, H). Additionally, Nissl staining revealed that HKL reduced neuronal loss and structural damage (Fig. 8I). TUNEL staining also showed that HKL inhibited I/R-induced apoptosis in mice (Fig. 8J, K). Notably, these neuroprotective effects of HKL were significantly enhanced by combining HKL with RIPC (Fig. 8I–K). Moreover, when compared with HKL alone, the combined therapy further suppressed oxidative stress after SCIRI, as revealed by higher antioxidant enzyme activity and lower MDA levels (Fig. 8L). These results indicate that HKL protects neurons from SCIRI in vivo and that its efficacy is improved by enhanced SIRT3 upregulation when combined with RIPC.

## Discussion

Remote ischemic conditioning, in which cycles of ischemia/reperfusion are applied to remote organ/tissue can stimulate a powerful innate mechanism of inter-organ protection [49]. Preclinical data and clinical trials indicate that RIPC therapy reduces myocardial injury and improves short-term outcomes in patients undergoing coronary artery bypass graft (CABG) surgery [50]. Meanwhile, RIPC can promote the release of molecules with damage-associated molecular pattern to activate natural defenses that protect the kidney against subsequent inflammatory and chemical injury [51]. In the central nervous system, RIPC prevents brain injury caused by hypothermic circulatory arrest (HCA) in a porcine model [10]. In this study, murine models were used to validate the protective role of limb RIPC in SCIRI. We performed five cycles of short-term ischemia–reperfusion on the ipsilateral limb one day before SCIRI, followed by behavioral and histological tests. Consistent with the findings of the majority of studies, our findings indicated that RIPC promoted the recovery of neurological function in SCIRI mice, inhibited oxidative stress in the spinal cord, and reduced neuronal damage. This demonstrated the reliability of the effect of RIPC against ischemic spinal cord injury.

Mitochondrial dysfunction and the release of ROS are critical and fundamental elements in the pathogenesis of I/R injuries [52]. Spinal cord cells are particularly susceptible to oxidative stress due to high metabolic activity, low levels of antioxidant enzymes and abundant cholesterol [53]. Accompanied by restoration of the blood supply, significant generation of ROS overwhelms the neutralization ability of tissues, resulting in enzyme inactivation, protein aggregation, lipid peroxidation, and a series of chain reactions [54]. Therefore, enhancing the antioxidant capacity of the spinal cord and preserving mitochondrial function are essential components of clinical treatment of SCIRI. Recent research indicates that RIPC can increase the activity of antioxidant enzymes in circulation and target organs [55]. Mitochondrial protein’s acetylation/deacetylation modification regulates mitochondrial function in response to redox derangements and cellular stress. Among the family of sirtuins, SITR3 is mainly expressed in the mitochondria and regulates redox homeostasis and other signaling pathways through deacetylation reactions [56]. Our results showed that SIRT3 expression decreased with reperfusion time following spinal cord ischemia; however, RIPC performed before ischemia increased SIRT3 expression and rescued SIRT3 downregulation induced by I/R. SIRT3 deficiency led to more severe neural damage in response to both I/R in vivo and OGD/R in vitro. This is attributed to the decreased activity of antioxidant enzymes regulated by SIRT3. More importantly, in Sirt3-KO mice, the protective effect of RIPC against SCIRI was attenuated, as was its inhibiting effect on oxidative stress. This emphasized the important role of SIRT3 in RIPC-mediated neuroprotection as a protein that functions downstream.

NMDAR is commonly typically characterized as two subtypes: synaptic NR2A and extra-synaptic NR2B two subtypes [57]. Currently, the role of these subtypes is contested, with some studies suggesting that NR2A promotes neuronal survival and NR2B causes neuronal death [58]. However, there is evidence to suggest that they play similar rather than opposing roles in NMDAR-mediated bidirectional regulation of pro-survival signaling and neuronal death [57]. Consistent with previous studies [12], we found that NR2B is involved in RIPC-mediated neuroprotection and that antagonizing NR2B significantly reduced the induction of SIRT3.

AMPK is an upstream signal of SIRT3 that regulates gene expression and the activity of PGC-1α [59], which induces SIRT3 expression by binding to estrogen-related receptor elements localized to the promoter region [60, 61]. AMPK phosphorylation is often inhibited following ischemic injury, resulting in inadequate cellular energy supply and redox imbalance [62]. The protective effect of the AMPK-PGC-1α-SIRT3 pathway against OGD/R-induced neuronal damage has been demonstrated [63]. RIPC induced increased AMPK activation in cerebral I/R injury, but inhibition of AMPK abrogates the neuroprotective effects [64]. Similarly, our present study observed that NMDA or RIPC treatment increased the activation of the AMPK-PGC-1α-SIRT3 pathway. In vitro, AMPK knockdown with shRNA or inhibition of AMPK activation with compound C suppressed NMDA-induced expression of PGC-1α and SIRT3. Compound C abolished the protective effect of NMDA pretreatment on neurons and the inhibitory effect of OGD/R-induced oxidative stress in cellular experiments. Therefore, we inferred that RIPC may improve spinal cord ischemic tolerance by upregulating SIRT3 expression through the AMPK-PGC-1α signaling pathway.

Although the majority studies have shown that SIRT3 is a downstream target gene for PGC-1α [60, 65, 66], emerging evidence suggests that SIRT3 deficiency affects AMPK phosphorylation and PGC-1α expression [67, 68]. RIPC was reported to up-regulate the expression of antioxidant factors such as nuclear factor (erythroid-derived 2)-like 2 (NRF2) and hypoxia-inducible factor 1α (HIF1α) [69, 70]. SIRT3 may act as an upstream regulator of these antioxidant factors, as both NRF2 and HIF1α are regulated by PGC-1α [71, 72]. Notably, it has been reported that the neuroprotection against glutamate cytotoxicity induced by NMDA preconditioning is mediated through activation of ERK1/2, inactivation of JNK, and prevention of glutamate-induced CREB inactivation [30, 73, 74]. However, the ERK-CREB axis has also been identified as the downstream effector of SIRT3 [75, 76]. SIRT3 downregulation is accompanied by decreased phosphorylation of AMPK and cAMP-response element-binding protein (CREB) in neurodegenerative disorders [77], indicating that SIRT3 could may indirectly regulate the ERK-CREB pathway. In light of the intricate interconnections between molecular signals, our study is not a contradiction, but rather a complement to the previous conclusions.

Ca^2+^ permeability of NMDARs enables them to trigger Ca^2+^-dependent signaling events. Notably, the level of cytosolic calcium (cCa^2+^) concentration affects Ca^2 +^ signaling [78]. Small amounts of calcium enter the mitochondria in favor of metabolic homeostasis, while large amounts are thought to induce cell death [79]. The phosphorylation of AMPK was significantly activated by transient elevations in cCa^2+^, while AMPK activity was blocked by persistently high levels of intracellular calcium [44, 80]. Similarly, after a short stimulation with sublethal concentrations of glutamate, intracellular calcium concentrations were moderately elevated, and the AMPK/PGC-1α/SIRT3 signaling pathway was activated. Continuous stimulation of neurons with lethal concentrations of glutamate, on the other hand, resulted in a significant increase intracellular calcium concentration and inhibition of the AMPK/PGC-1α/SIRT3 signaling pathway. Before stimulation of neurons with lethal doses of glutamate, BAPTA, an intracellular calcium chelator, significantly reversed the activity of AMPK, demonstrating the biphasic effect of intracellular calcium concentration on AMPK activity. To further investigate why lethal doses of glutamate inhibit AMPK phosphorylation, we first examined the altered activation of the upstream kinase CaMKKβ, which has been reported to phosphorylate AMPK on threonine 172 in a Ca^2+^ dependent manner [81], resulting in enhanced AMPK catalytic activity [82]. Contrary to expectations, we found that, like sublethal does of glutamate, lethal doses of glutamate also promoted the activation of CaMKKβ, suggesting that the inhibition of AMPK phosphorylation by persistently elevated intracellular Ca^2+^ was not mediated by CaMKKβ. Delayed clearance of cCa^2+^ in the liver was found to result in elevated Ca^2+^-dependent PP4 activity in the cytoplasm, resulting in increased AMPKα dephosphorylation [44]. Therefore, we hypothesized that sustained high levels of cCa^2+^ within neurons could inhibit AMPK phosphorylation by increasing PP4 activity. We examined changes in PP4 expression in neurons following treatment with lethal doses of glutamate and found that PP4 expression increased with time in neurons. After inhibiting PP4 with cantharidin, the inhibitory effect of toxic doses of glutamate on AMPK/PGC-1α/SIRT3 signaling was significantly abolished. Therefore, the inhibition of the AMPK/PGC-1α/SIRT3 signaling pathway induced by lethal doses of glutamate may be caused by an increase in AMPK dephosphorylation due to the sustained high levels of cCa^2+^ activating PP4. The use of PP4 inhibitors to improve spinal cord ischemia tolerance may be a promising approach in the future.

Our findings above indicate that SIRT3 plays an important role in SCIRI. Therefore, we also investigated the therapeutic effects of SIRT3 agonists on SCIRI. HKL [2-(4-hydroxy-3-prop-2-enyl-phenyl)-4-prop-2-enyl-phenol] is a natural bisphenol compound with a small molecular weight that is derived from the bark of magnolia trees and used in Asian traditional medicine systems [83]. HKL is a natural SIRT3 pharmacological agonist that not only induces SIRT3 expression but also enters the mitochondria and binds directly to SIRT3 to increase its deacetylase activity [48]. HKL possesses potent antioxidant and anti-inflammatory properties and may readily cross the blood–brain barrier and the blood-spinal cord barrier, exerting neuroprotective effects via a variety of mechanisms [84]. HKL is reported to have a protective effect against ischemia–reperfusion injury in a variety of organs, including the heart, brain, and kidneys [85–87]. In addition, HKL can impede the neurotoxic calcium influx through NMDA receptor channels and play a protective role in cerebral ischemia reperfusion injury [87]. However, few studies have investigated its effect on the spinal cord. Our findings demonstrate that HKL has neuroprotective effects in vitro, inhibiting OGD/R-induced neuronal damage and reducing oxidative stress. In addition, we discovered that HKL application after OGD/R significantly increased SIRT3 expression by inhibiting NMDAR hyperactivation and partially restoring AMPK/PGC-1α signaling pathway activation. Although the NMDA receptor plays an important role in glutamate-induced excitotoxicity in ischemia conditions, almost all NMDA receptor antagonists failed to reduce the human brain's vulnerability to ischemia in clinical trials [88]. In a study of NMDA-induced brain injury, HKL, but not NMDAR antagonist meperidine, was observed exert a protective effect after injury alone [89]. Therefore, in this study, we used HKL rather than memantine as post-I/R therapeutic agents. Nonetheless, the clinical value of combining NMDAR antagonists with drugs acting through other different mechanisms such as HKL, following I/R warrants further investigation in the future, and the feasibility of this hypothesis has been confirmed by several studies [89].

Due to the limitations of monotherapy, combination therapy is becoming the mainstay of clinical treatment [90, 91]. Combination therapy is a new, more global strategy to increase the potential benefits with the combined use of already available disease-modifying therapies or with other new agents that have an acceptable theoretical rationale and good safety and efficacy profile [44]. Few studies, however, have reported on the use of combination therapy regimens in the field of SCIRI. This may be due to the complexities of clinical application and the mechanisms of interference between the various treatment modalities. Given the benefits of RIPC and HKL for SCIRI, the temporal mismatch between the two regimens, and the similar protective mechanisms, we propose a SIRT3-centered combination regimen for SCIRI. The regimen is highly clinically tractable, that is, RIPC is performed before thoracoabdominal aortic or spinal cord decompression surgery to improve spinal cord ischemic tolerance by promoting SIRT3 expression, and continuous SIRT3 agonist administration after surgery to further inhibit oxidative stress and reduce neurological injury. Our findings imply that a combination of pre-conditioning and post-conditioning provides synergistic protection against spinal cord ischemia reperfusion injury, which will lead to the development of novel approaches and treatment options for SCIRI in the future.

## Conclusion

**Fig. 9:**
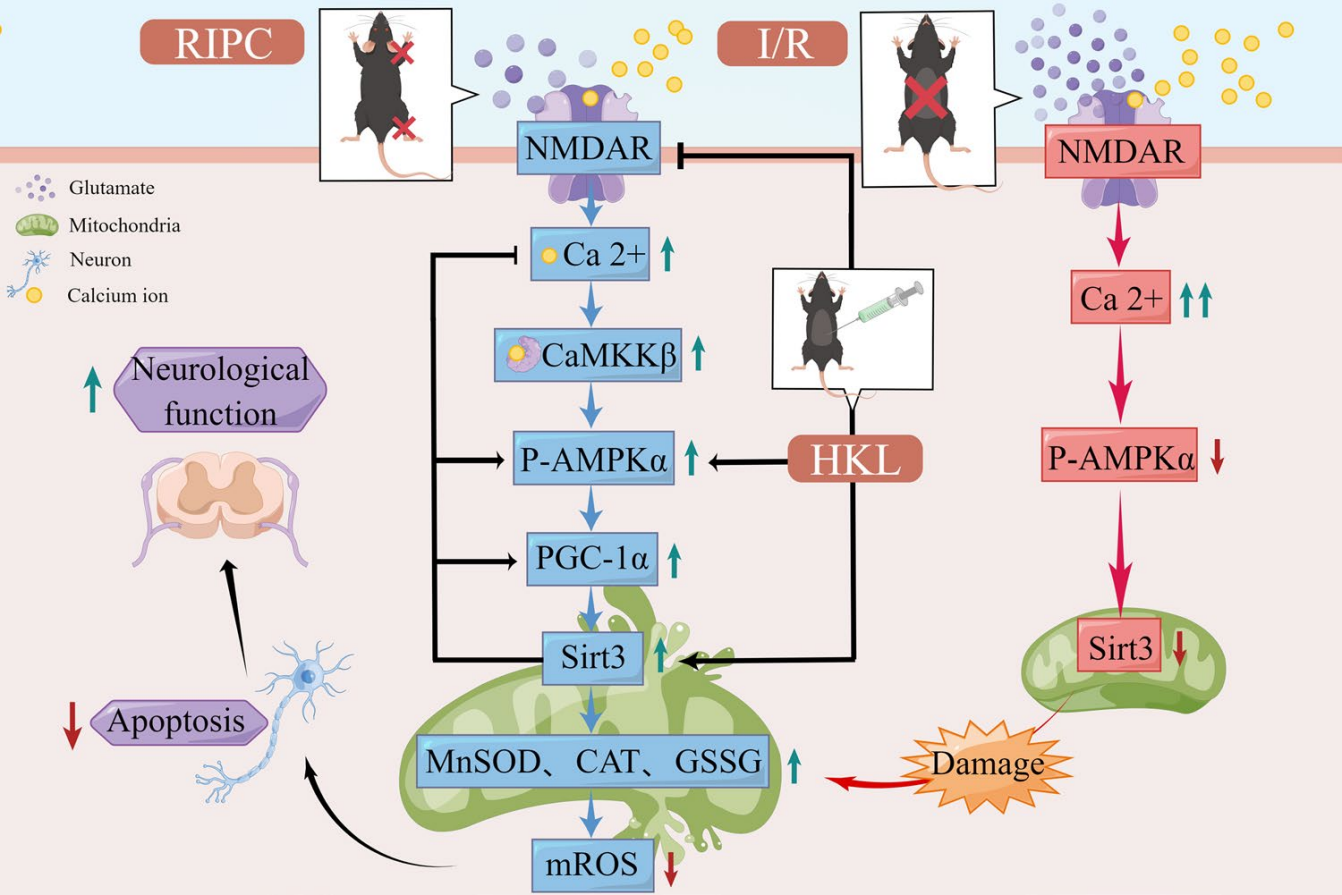
Molecular mechanisms of the three different treatments of RIPC, I/R and HKL administration. RIPC: Sub-lethal remote ischemic preconditioning activates NMDAR2B receptors on the neuronal surface by slightly up-regulating glutamate concentrations outside the spinal anterior horn motor neurons, leading to a transient mild increase in intracellular Ca^2+^ concentration, activation of the AMPK-PGC-1α signaling pathway via CaMKKβ, increase in mitochondrial SIRT3 expression, and improved neuronal ischemic tolerance. *I/R:* Lethal ischemia causes an extreme increase in glutamate concentration and excessive activation of NMDAR, resulting in sustained high levels of Ca^2+^ in neurons, inhibiting AMPK activation, down-regulation of mitochondrial SIRT3 expression, and generation of large amounts of ROS leading to neuronal apoptosis. *HKL*: HKL improves spinal cord antioxidant capacity after I/R by inhibiting NMDAR hyperactivation and increasing SIRT3 activity. HKL combined with RIPC better promotes neurological recovery in mice after SCIRI

This study demonstrates for the first time that RIPC inhibits mitochondrial oxidative stress and is resistant to SCIRI by regulating SIRT3 expression via the NMDAR/AMPK/PGC-1α signaling pathway. It contributes to a deeper understanding of the neuroprotective mechanisms of RIPC. In addition, we demonstrate that the use of SIRT3 agonists HKL following SCIRI has a neuroprotective effect. The combination of SIRT3 agonist and RIPC has a synergistic effect and better promotes the recovery of spinal cord neurological function in mice. This is the first combination therapy regimen proposed for SCIRI treatment based on SIRT3 (Fig. 9).

## Supplementary Information

**Additional file 1: Fig. S1.** RT-PCR genotyping with primers for SIRT3 WT and KO. **Fig. S2.** Immunocytochemical identification of primary neurons. Neuronal dendrites and axons were identified by anti-MAP2 (green) and somata by NeuN (red) immunostaining. The nuclei of all cells were identified by DAPI (blue). Scale bar = 100μm. **Fig. S3.** Toxic concentrations of glutamate did not inhibit the phosphorylation of CaMKKβ. Immunoblot analysis for phospho and total CaMKKβ and quantification of the ratio of phospho CaMKKβ to total CaMKKβ (n = 5/group). Statistical analysis: mean ± SEM. *p < 0.05, **p < 0.01, ***p < 0.001. **Fig. S4.** SIRT3 deficiency attenuates the neuroprotective effect of HKL on SCIRI mice. A BMS scores at different time points post-injury in WT or KO mice treated with or without HKL (n=5/group). B Representative footprint images of WT or KO mice treated with or without HKL on day 3 after I/R. Blue: frontpaw print; red: hindpaw print. C Quantitative analysis of the footprint in figure B (n=5/group). D Representative images of MEP for assessing the electrophysiology of WT or KO mice treated with or without HKL on day 3 after I/R. E Quantification of the peak-to-peak MEP amplitudes in figure D (n=5/group). F Representative images of Nissl staining of neurons in the anterior horn of the spinal cord. Scale bar = 100μm. G Quantification of the number of integrated Nissl bodies per section (n=5/group). H Representative images of TUNEL-positive apoptotic cells (in red) in spinal cord sections on day 3 post-injury. Neuron was stained with NeuN (in green) and nuclear stained with DAPI (in blue). Scale bar = 100μm. I Quantification of the number of apoptotic cells in each group (n=5/group). J MDA, MnSOD, CAT, and GSH were measured to reflected the level of oxidative stress in each group (n=5/group). Statistical analysis: mean ± SEM, *p < 0.05, **p < 0.01, ***p < 0.001. **Fig. S5.** Original immunoblot pictures of all the mice used.

## Data Availability

The datasets used and/or analyzed during the current study are available from the corresponding author on reasonable request.
