# Supplementary material for: Remote ischemic preconditioning protects against spinal cord ischemia–reperfusion injury in mice by activating NMDAR/AMPK/PGC-1α/SIRT3 signaling
Source: Cell Biosci. 2023 Mar 16;13:57. doi: 10.1186/s13578-023-00999-4 (PMC10018930; doi:10.1186/s13578-023-00999-4)
Supplement: Supplementary file 1 — Additional file 1: Fig. S1. RT-PCR genotyping with primers for SIRT3 WT and KO. Fig. S2. Immunocytochemical identification of primary neurons. Neuronal dendrites and axons were identified by anti-MAP2 (green) and somata by NeuN (red) immunostaining. The nuclei of all cells were identified by DAPI (blue). Scale bar = 100μm. Fig. S3. Toxic concentrations of glutamate did not inhibit the phosphorylation of CaMKKβ. Immunoblot analysis for phospho and total CaMKKβ and quantification of the ratio of phospho CaMKKβ to total CaMKKβ (n = 5/group). Statistical analysis: mean ± SEM. *p < 0.05, **p < 0.01, ***p < 0.001. Fig. S4. SIRT3 deficiency attenuates the neuroprotective effect of HKL on SCIRI mice. A BMS scores at different time points post-injury in WT or KO mice treated with or without HKL (n=5/group). B Representative footprint images of WT or KO mice treated with or without HKL on day 3 after I/R. Blue: frontpaw print; red: hindpaw print. C Quantitative analysis of the footprint in figure B (n=5/group). D Representative images of MEP for assessing the electrophysiology of WT or KO mice treated with or without HKL on day 3 after I/R. E Quantification of the peak-to-peak MEP amplitudes in figure D (n=5/group). F Representative images of Nissl staining of neurons in the anterior horn of the spinal cord. Scale bar = 100μm. G Quantification of the number of integrated Nissl bodies per section (n=5/group). H Representative images of TUNEL-positive apoptotic cells (in red) in spinal cord sections on day 3 post-injury. Neuron was stained with NeuN (in green) and nuclear stained with DAPI (in blue). Scale bar = 100μm. I Quantification of the number of apoptotic cells in each group (n=5/group). J MDA, MnSOD, CAT, and GSH were measured to reflected the level of oxidative stress in each group (n=5/group). Statistical analysis: mean ± SEM, *p < 0.05, **p < 0.01, ***p < 0.001. Fig. S5. Original immunoblot pictures of all the mice used. [file 13578_2023_999_MOESM1_ESM.docx]

**Additional materials**


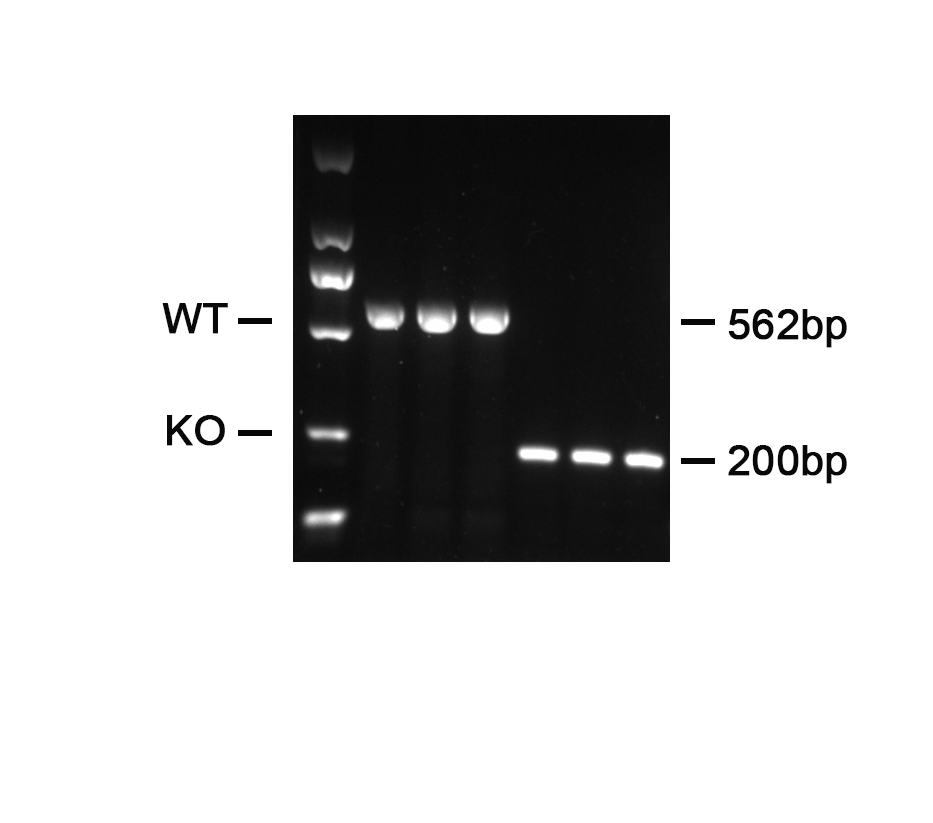


**Fig. S1 RT-PCR genotyping with primers for SIRT3 WT and KO**

A 562-bp fragment was generated from the WT allele, and a 200-bp fragment from the KO allele.


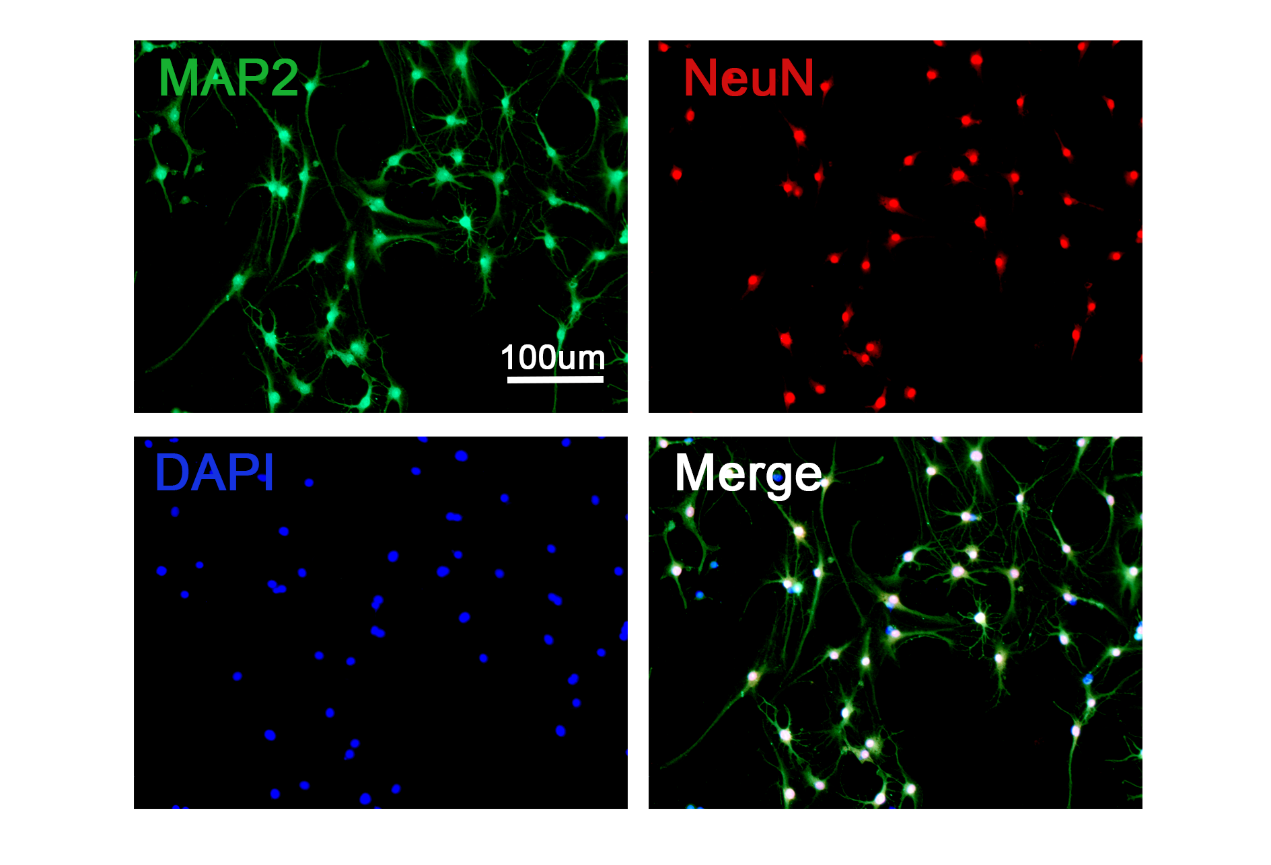


**Fig. S2 Immunocytochemical identification of primary neurons.**

Neuronal dendrites and axons were identified by anti-MAP2 (green) and somata by NeuN (red) immunostaining. The nuclei of all cells were identified by DAPI (blue). Scale bar = 100μm.


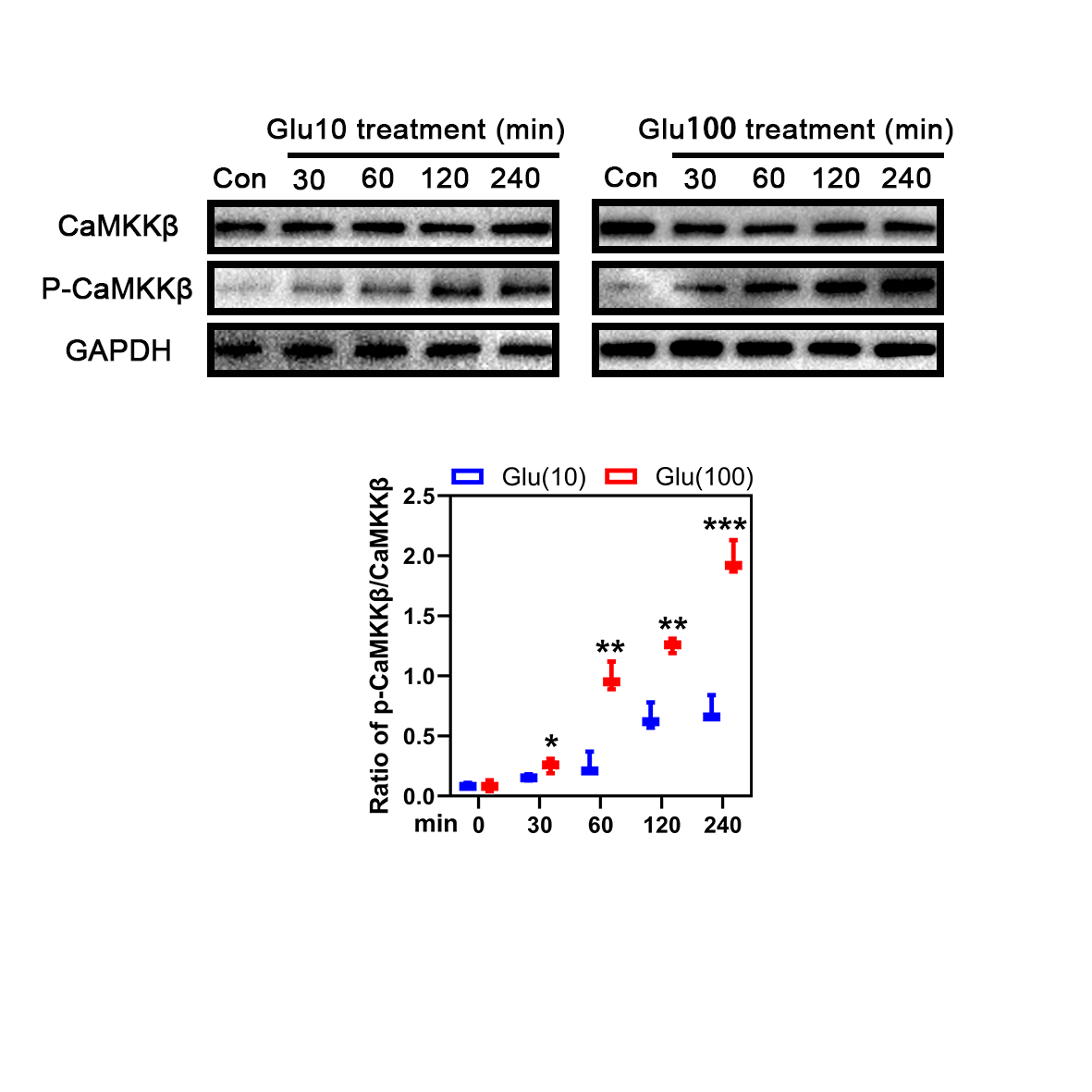


**Fig. S3 Toxic concentrations of glutamate did not inhibit the phosphorylation of** **CaMKKβ**

Immunoblot analysis for phospho and total CaMKKβ and quantification of the ratio of phospho CaMKKβ to total CaMKKβ (n = 5/group). Statistical analysis: mean ± SEM. *p < 0.05, **p < 0.01, ***p < 0.001.


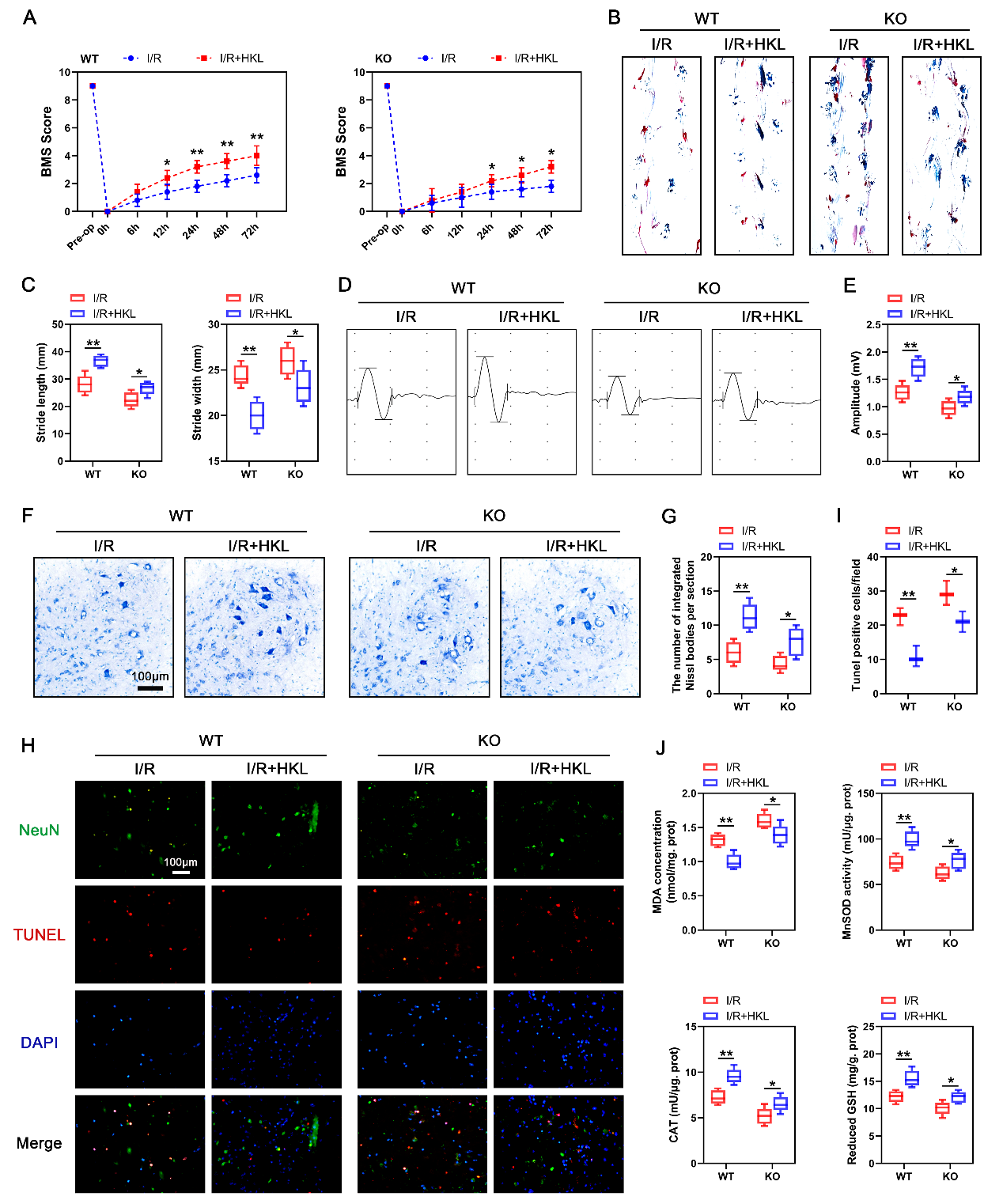


**Fig. S4 SIRT3 deficiency attenuates the neuroprotective effect of HKL on SCIRI mice**

**A** BMS scores at different time points post-injury in WT or KO mice treated with or without HKL (n=5/group).

**B** Representative footprint images of WT or KO mice treated with or without HKL on day 3 after I/R. Blue: frontpaw print; red: hindpaw print.

**C** Quantitative analysis of the footprint in figure B (n=5/group).

**D** Representative images of MEP for assessing the electrophysiology of WT or KO mice treated with or without HKL on day 3 after I/R.

**E** Quantification of the peak-to-peak MEP amplitudes in figure D (n=5/group).

**F** Representative images of Nissl staining of neurons in the anterior horn of the spinal cord. Scale bar = 100μm.

**G** Quantification of the number of integrated Nissl bodies per section (n=5/group).

**H** Representative images of TUNEL-positive apoptotic cells (in red) in spinal cord sections on day 3 post-injury. Neuron was stained with NeuN (in green) and nuclear stained with DAPI (in blue). Scale bar = 100μm.

**I** Quantification of the number of apoptotic cells in each group (n=5/group).

**J** MDA, MnSOD, CAT, and GSH were measured to reflected the level of oxidative stress in each group (n=5/group).

Statistical analysis: mean ± SEM, *p < 0.05, **p < 0.01, ***p < 0.001.


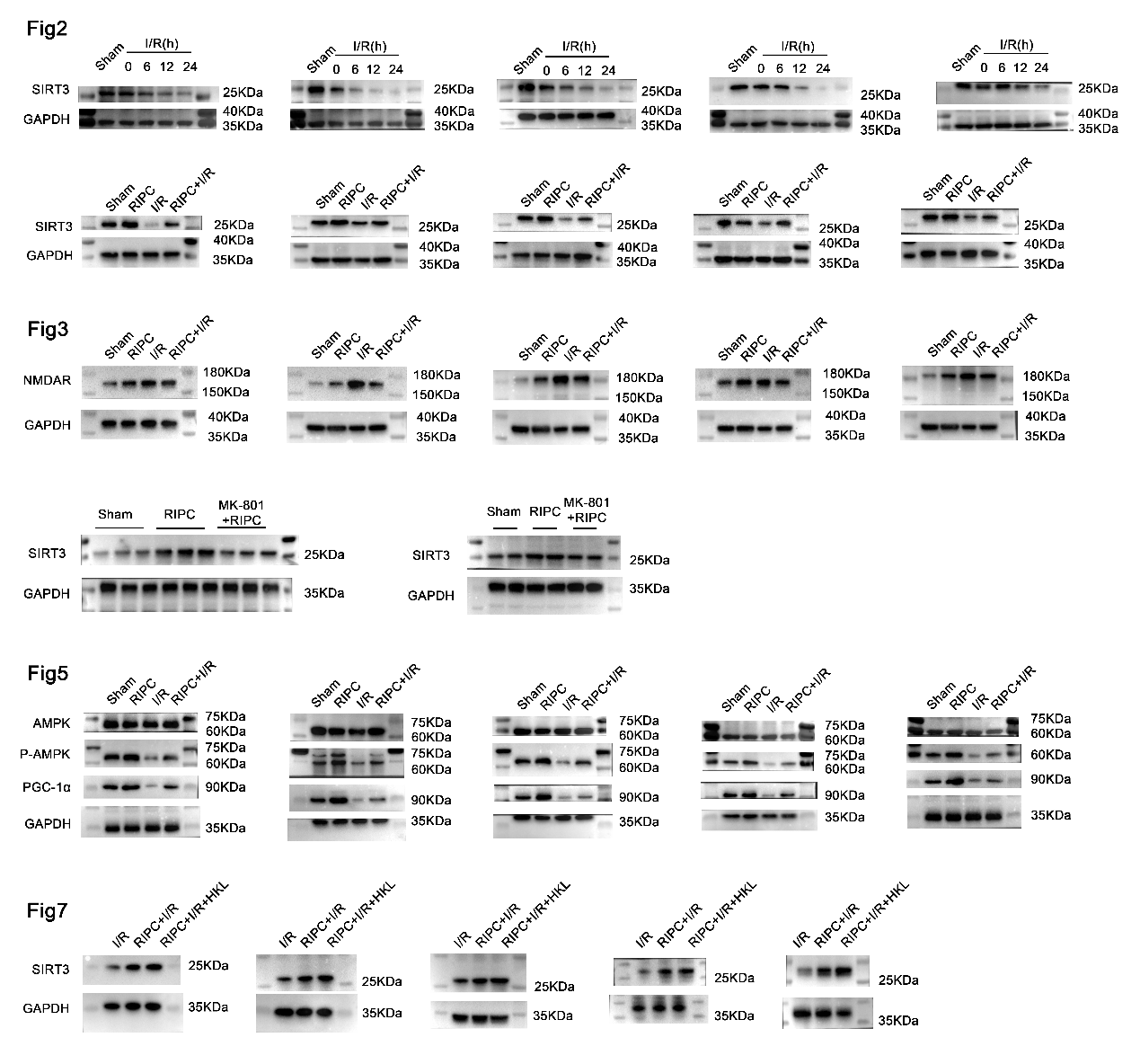


**Fig. S5** **Original immunoblot pictures of all the mice used**
